# MIF-expressing tumor cells mediate immunotherapeutic resistance in esophageal squamous cell carcinoma

**DOI:** 10.7150/thno.118269

**Published:** 2026-01-01

**Authors:** Jing Song, Xiaomei Song, Yue Xie, Hong Guo, Yupeng Cun

**Affiliations:** 1 Pediatric Research Institute, Ministry of Education Key Laboratory of Child Development and Disorders, Chongqing Key Laboratory of Cognitive Development and Learning and Memory Disorders, National Clinical Research Center for Child Health and Disorders, Children's Hospital of Chongqing Medical University, No. 136 Zhongshan 2nd Road, Yuzhong District, 400014, Chongqing, China.; 2 Department of Gastroenterology, Chongqing General Hospital, Chongqing University, 118 Xingguang Avenue, Yubei District, 401147, Chongqing, China.

**Keywords:** esophageal squamous cell carcinoma, immunotherapy, B cell, cholesterol biosynthesis, ScRNA-seq, spatial RNA sequencing

## Abstract

**Background**: Despite the use of immunotherapy in esophageal squamous cell carcinoma (ESCC), treatment failure occurs occasionally in patients, yet the underlying mechanisms remain poorly understood.

**Methods**: We conducted large-scale single-cell RNA sequencing (scRNA-seq) data analysis, which integrated seven independent datasets from 192 ESCC patients to yield over 440,000 high-quality single cells, to systematically characterize the tumor microenvironment (TME) landscape during ESCC progression and immunotherapy response. Additionally, we performed high-resolution spatial transcriptomics (stRNA-seq) using the 10x Visium HD platform on paired pre- and post-treatment tissues from two patients (one immunotherapy responder and one non-responder), which enhanced the findings from the scRNA-seq data and mapped therapy-induced TME at the spatial level. Multiplex immunohistochemistry was employed based on seven patients to confirm distinct patterns of intercellular crosstalk underlying differential therapeutic outcomes.

**Results**: In scRNA-seq data, we found that B lineage cells were reduced during ESCC progression but were enriched in immunotherapy-resistant patients. Further analysis of malignant ESCC cells suggested that immunotherapy resistance might be associated with a subpopulation of tumor cells exhibiting aberrantly elevated cholesterol biosynthesis. Cell communication analysis of scRNA-seq and stRNA-seq data collectively revealed that immunotherapy resistance was linked to cellular crosstalk between cholesterol-biosynthetic tumor cells and germinal center (GC) B cells within tertiary lymphoid structures. Notably, single-cell, spatial data, and multiplex immunohistochemistry demonstrated that cholesterol biosynthesis-associated ESCC cells express elevated levels of MIF. This disrupts GC reactions by competing with the CXCL12-CXCR4 signaling axis via MIF-CXCR4 interactions, thereby impairing B cell-mediated immunity.

**Conclusions**: MIF^+^ tumor cells in GCs may be a biomarker for predicting immunotherapy resistance in ESCC.

## Introduction

According to GLOBOCAN statistics, over 600,000 new cases of esophageal cancer were reported globally in 2020, with approximately 510,000 (~85%) classified as esophageal squamous cell carcinoma (ESCC) 1. Eastern Asia bears the highest incidence and mortality burden, with a male-to-female ratio of ~2:1 among newly diagnosed cases. The five-year survival rate remains below 30%, mainly due to frequent recurrence and drug resistance 1. Platinum-based chemotherapy is the standard first-line regimen for advanced ESCC 2. Notably, the phase III JUPITER-06 trial demonstrated that combining platinum-paclitaxel (TP) chemotherapy with anti-PD-1 therapy significantly improved outcomes in locally advanced or metastatic ESCC 2. However, 26.8% (69/257) of patients exhibited stable or progressive disease 2, highlighting the urgent need to decipher molecular mechanisms of the tumor microenvironment (TME) underlying immunotherapy resistance. While tertiary lymphoid structures (TLS) are predictive biomarkers for immunotherapy response, ~20-30% of TLS-rich tumors paradoxically resist treatment 3, suggesting functional heterogeneity within TLS that remains mechanistically undefined.

TLS are ectopic lymphoid aggregates that form in non-lymphoid tissues during chronic inflammation or cancer 4, 5. Structurally composed of B cells, T cells, dendritic cells, and stromal networks, TLS actively coordinate local antigen presentation and adaptive immune responses 4, 5. Germinal centers (GCs), critical functional compartments within TLS, are sites where antigen-activated B cells undergo clonal proliferation and affinity maturation - processes essential for their differentiation into either plasma cells (antibody-secreting effectors) or memory B cells (mediators of secondary immunity) 6. Recent studies have established a strong association between GC B cells and immunotherapy sensitivity in ESCC 7. However, the mechanisms underlying this association remain poorly defined, as evidenced by the paradoxical coexistence of TLS and immunotherapy resistance (e.g., TLS-positive tumors refractory to treatment) 3. To address this gap, longitudinal profiling of the TME in paired pre-/post-treatment samples, with spatial resolution mapping of TLS niches and immune dynamics, could elucidate how GC B cells modulate TLS-driven immunotherapy sensitivity in ESCC.

Through integrative analysis of spatial transcriptomics in two paired pre-/post-treatment ESCC specimens (n = 2 patients) combined with single-cell RNA-seq (scRNA-seq) data from ~440,000 high-quality cells, we identified that MIF-expressing tumor cells competitively disrupted CXCL12-CXCR4 signaling in GC B dark-zone cells via direct MIF-CXCR4 interactions, thereby impairing GC reactions within the TME. Notably, these findings suggest that MIF^+^ tumor cells infiltrating TLS, rather than TLS status alone, may serve as a co-factor for evaluating immunotherapy response, a phenomenon detectable even in baseline tumors of ESCC non-responders. Furthermore, multiplex immunohistochemistry (mIHC) analysis of three paired pre-/post-treatment ESCC tissues and four additional pre-treatment tumor samples confirmed that MIF-high tumor cells spatially interacted with GC B cells in non-responder tumors. Collectively, our study systematically characterizes the ESCC TME landscape, leverages longitudinal specimens to identify immunotherapy resistance-associated cellular markers, and provides actionable insights for developing novel therapeutic strategies to overcome resistance and enhance clinical outcomes in ESCC.

## Materials and Methods

### Patient Cohort and Sample Characteristics

This study analyzed tumor specimens from 7 ESCC patients who underwent TP chemotherapy combined with anti-PD-1 therapy (camrelizumab or sintilimab) between January 2023 and January 2025. This included 6 specimens from 3 patients (2 responders, 1 non-responder) collected both pre- and post-treatment (forming 3 pairs) specimens, and 4 pre-treatment specimens from additional 4 patients (3 responders, 1 non-responder). All pre-treatment samples were obtained two weeks prior to treatment. All specimens were collected from Chongqing General Hospital with ethical approval by Medical Ethics Committee of Chongqing General Hospital (IIT S2025-008-01). Written informed consent was obtained from all patients (n = 7). Formalin-Fixed and Paraffin-Embedded (FFPE) slides were shipped for sequencing (August 3, 2024). Patient characteristics and clinical information are shown in Table S1.

#### Responders (Patient 1, patient 3, Patient 5-7)

Patient-1: 79-year-old male, non-smoker/non-drinker, stage IIIB (pT3N1M0) at diagnosis. Paired samples (pre-treatment and post-treatment) underwent Visium HD spatial transcriptomics (RNA Integrity Number/RIN: 2.2-2.3) and mIHC, confirming partial response (PR) based on RECIST v1.1.

Patient-3: 75-year-old female, non-smoker/non-drinker, stage IIB (pT1N1M0). Pre-treatment (RIN = 3.3) and post-treatment (RIN = 3.9) samples were analyzed.

Patient 5-7: 73-77 years old, male, non-smokers/non-drinkers. Only pre-treatment sample was available and was analyzed using mIHC.

#### Non-responders (Patient-2 and patient-4)

Patient-2: A 53-year-old male, non-smoker/non-drinker, was initially diagnosed with stage IVB (pT2N0M1) with post-treatment progression. Paired samples (pre-treatment and post-treatment) were subjected to Visium HD sequencing (RIN: 2.2-2.5) and mIHC, consistent with progressive disease (PD).

Patient-4: A 74 year-old female, non-smoker/non-drinker, was diagnosed with stage IVB (pT3N2M1) with post-treatment progression. Only pre-treatment sample was available and was analyzed using mIHC.

### Spatial transcriptome sequencing and processing

Spatial transcriptome sequencing of four FFPE tissues of two ESCC patients with pre- and post-immunotherapy was conducted using the 10x Genomics Visium HD platform, which offered a resolution of 2 μm × 2 μm. Quality assessment and sequencing of tissue sections were performed. First, SpaceRanger (v3.1.0) was utilized for aligning FASTQ files to human reference genome (GRCh38), detecting tissue sections, and mapping sequencing data to both microscope and CytAssist images. Gene-barcode matrices were aggregated into 8 μm × 8 μm binned squares for subsequent analyses. According to a recent benchmarking study 8, Robust Cell Type Decomposition (RCTD) demonstrated consistently high performance when using external reference datasets. Given that our study utilizes an integrated single-cell dataset as an external reference, RCTD was shown to be the best-performing method under such conditions. Thus, cell type deconvolution was conducted using RCTD algorithm in spacexr (v2.2.1) 9, and run in doublet mode. Pre-annotated scRNA-seq data were used as the reference dataset. Squares containing fewer than 10 detected features were excluded from analysis.

### ScRNA-Seq data and preprocessing

ScRNA-Seq (10x Genomics) data of 192 ESCC patient tissues were retrieved from five publicly available datasets: GSE145370 10, GSE160269 11, GSE199654 12, GSE203115 13, and GSE221561 14. Another two datasets of normal esophageal cells (GSE196756 15, GSEE188900 16) were severed as normal control also integrated to the aforementioned five ESCC datasets. Patients with no clinical information were excluded. Patient characteristic, sample information, number of cells pre- and post-filter were summarized in Table S2. The count expression matrices of scRNA-seq were merged via Seurat *merge* function (v4.3.1) 17. Genes expressed in fewer than three cells were filtered out. To exclude low-quality cells, we applied stringent filtering criteria, removed cells with nFeatures_RNA < 200, nCount_RNA < 500, or mitochondrial gene content > 20%. Cell clustering was performed using Seurat with default parameters. We employed FastMNN algorithm in SeuratWrappers (v0.3.1) to remove batch effect among seven datasets. Uniform manifold approximation and projection (UMAP) embeddings were computed based on top 30 principal components and *mnn* reduction. Cell clusters were identified using the Louvain algorithm with a resolution parameter of 0.1 18, 19. At higher resolution values (e.g., 0.5 or 1.0), the clustering results produced excessive fragmentation of known cell types (e.g., splitting well-defined immune subsets such as naive and memory B cells into multiple small clusters), whereas resolution = 0.1 yielded clusters that more accurately corresponded to canonical cell types, as supported by marker gene expression. Then, doublets were detected and filtered using scDblFinder (v1.8.0) 20, with doublet ratio set to 0.76. Clusters exhibiting mixed lineage markers (e.g., co-expression of *CD3* and *EPCAM*) were also classified as doublets and excluded from further analyses.

### Cell type annotation and trajectory analysis in scRNA-seq data

Cell types were annotated based on curated marker genes (Table S3). For the major cell types, the following markers were used: *EPCAM, KRT14, TP63* for epithelial cells; *KRT4, KRT13, GJB2* for basal cells; *DCN*, *ACTA2*, *FBLN1* for fibroblasts; *PECAM1*, *ENG*, *PLVAP* for endothelial cells; *CD3D*, *CD3E*, *CD8A*, *NKG7* for T/NK cells; *CD68*, *CD14*, *FCGR3A* for myeloid cells; *CD19*, *MS4A1*, *BANK1* for B cells; *MZB1*, *JCHAIN*, *DERL3* for plasma cells; and *FCER1A*, *TPSB2*, *CPA3* for mast cells. Subsets of cell populations were identified by re-clustering each major cell type using the same methods described above. For B cells, the following markers were used for annotation: *IGHM*, *IGHD* for naive B cells; *CD80*, *CD82* for activated B cells; *BANK1* for memory B cells; *CXCR4*, *STMN1*, *TOP2A*, *MKI67*, and negative expression of *CD83* and *CD86* for germinal center (GC) B dark zone cells; and *CXCR5*, positive *CD83*, and positive *CD86* for GC B light zone cells.

To delineate B cell differentiation dynamics, we performed single-cell trajectory inference using the slingshot R package (v2.8.0) 21. The Seurat object containing B cells was converted to a SingleCellExperiment object using the raw counts and cell subtype annotations. A set of 2,000 highly variable genes were utilized for trajectory construction. The starting point of the trajectory was defined as the naive B cell subpopulation. Cell embeddings derived from the MNN-corrected data were used to generate UMAP plots, ensuring consistency with the cell annotations.

### Inferring copy number profile in scRNA-seq data

Malignant epithelial cells in scRNA-seq profile of ESCC were identified by inferCNV (v1.10.0) 22. Immune cells were used as reference cell types. Copy number variation (CNV) scores of cells were calculated as the sum of absolute values of scaled CNV estimations. Scaled CNV scores > 0.04 were determined as potential malignant cells based on previous literature 23.

### Non-negative matrix factorization (NMF) and module analysis in scRNA-seq

NMF (v0.26) (http://renozao.github.io/NMF/) was used to identify functional modules in epithelial cells of ESCC. Here, samples with cell numbers <100 were filtered in this analysis. *nsNMF* method was applied for ranks between 5 and 15. Module name of each module was assigned based on functional enrichment of module genes via *g:Profiler* web tool. These module scores of each module in samples were calculated using the module gene expression based on *AddModuleScore* implemented in the Seurat.

Cholesterol biosynthesis-related malignant cells were identified by activated cholesterol biosynthesis score bigger than 0. Differential expression genes between cholesterol biosynthesis-related cells and other malignant cells were calculated by *FindMarkers* of the Seurat. These marker genes expressed in cholesterol biosynthesis-related cells (pct.1) and other malignant cells (pct.2) were used to evaluate the specificity (pct.1 divided by pct.2) of gene expression in different cell groups.

### Cell-cell communications

Cell-cell communication analysis was performed using scRNA-seq data via CellChat (v2.0) 24. Colocalization of a ligand-receptor pair was defined by co-expression (count > 1) of ligands and receptors in the same square of the spatial RNA-seq data. We were primarily interested in potential communications between cholesterol biosynthesis-related tumor cells and GC B cells.

### Bulk RNA-Seq data analysis of ESCC for survival and therapy response

Gene signatures for cholesterol biosynthesis were calculated as sum of the scaled expression levels of the associated marker genes. For Kaplan-Meier survival analysis, median value of the signature scores of cholesterol biosynthesis-related cell signature was used as cut-off value to stratify patients into high-score and low-score groups. Survival differences were assessed by log-rank test. Patients with missing outcomes (e.g., deceased or progressed) or missing follow-up data were excluded from survival analysis. To evaluate the difference in signature scores of cholesterol biosynthesis-related cells between responder and non-responder patients who underwent immunotherapy, IMvigor210 cohort (n = 298) 25 was analyzed. The IMvigor210 cohort comprised 298 patients with locally advanced or metastatic urothelial cancer (male/female = 233/65; median age 67 years) treated with atezolizumab (anti-PD-L1). The majority were White (270/298), with 7 Asian, 9 Black/African American, and 12 of other/unknown race. 233 patients had received prior platinum-based therapy. Analysis of IMvigor210 cohort was to investigate whether the molecular signature of cholesterol biosynthesis might be broadly associated with immunotherapy resistance across cancers. Two-tailed Mann-Whitney U test was used to compare signature scores between responders and non-responders.

### Hematoxylin and eosin (H&E) and immunohistochemistry (IHC) staining

FFPE human tissue sections were processed following standard protocols. Sections of 5-μm thickness were used for staining. The steps of H&E and IHC staining were described previously 26. Primary antibodies against HMGCR (Huabio, ET1702-41, 1:150 dilution) and MIF (Cell Signaling Technology, 87501, 1:150 dilution) were used. Results were visualized and imaged using a bright-field microscope.

### Multiplex immunohistochemistry (mIHC)

FFPE tissue sections (5-μm thickness) of two ESCC patients were used for mIHC. Slides were incubated with primary antibodies against HMGCR (Huabio, ET1702-41, 1:300 dilution), CXCR4 (Huabio, HA722304, 1:250 dilution), CD83 (Huabio, ER62949, 1:250 dilution), CD86 (Huabio, ER1906-01, 1:250 dilution), pan-Cytokeratin (pan-CK, Abcam, ab7753, 1:250 dilution), MIF (Cell Signaling Technology, 87501, 1:250 dilution), CD3 (Proteintech, 17617-1-AP, 1:300), CD20 (Proteintech, 60271-1-Ig, 1:300), CD21 (Proteintech, 24374-1-AP, 1:300), CD27 (Proteintech, 66308-1-Ig, 1:300), PD-1 (Proteintech, 18106-1-AP, 1:300), TIM-3 (Proteintech, 11872-1-AP, 1:300), SOX2 (Proteintech, 66411-1-Ig, 1:250) overnight at 4 ℃. Tyramide Signal Amplification Kit (RecordBio Co. Ltd, RC0086Plus-67RM) was used in this assay. Antibodies were validated using tissue slides in this study.

### Statistics analysis

Survival analysis was performed using log-rank test. Comparison of signature scores was performed using two-tailed Mann-Whitney U test. For functional enrichment analysis of genes, hypergeometric test was used. All analyses were performed using R software (v4.3.3).

## Results

### Tumor microenvironment landscape reveals a positive role for B cells in anti-tumor progression and immunotherapy response

**Figure 1:**
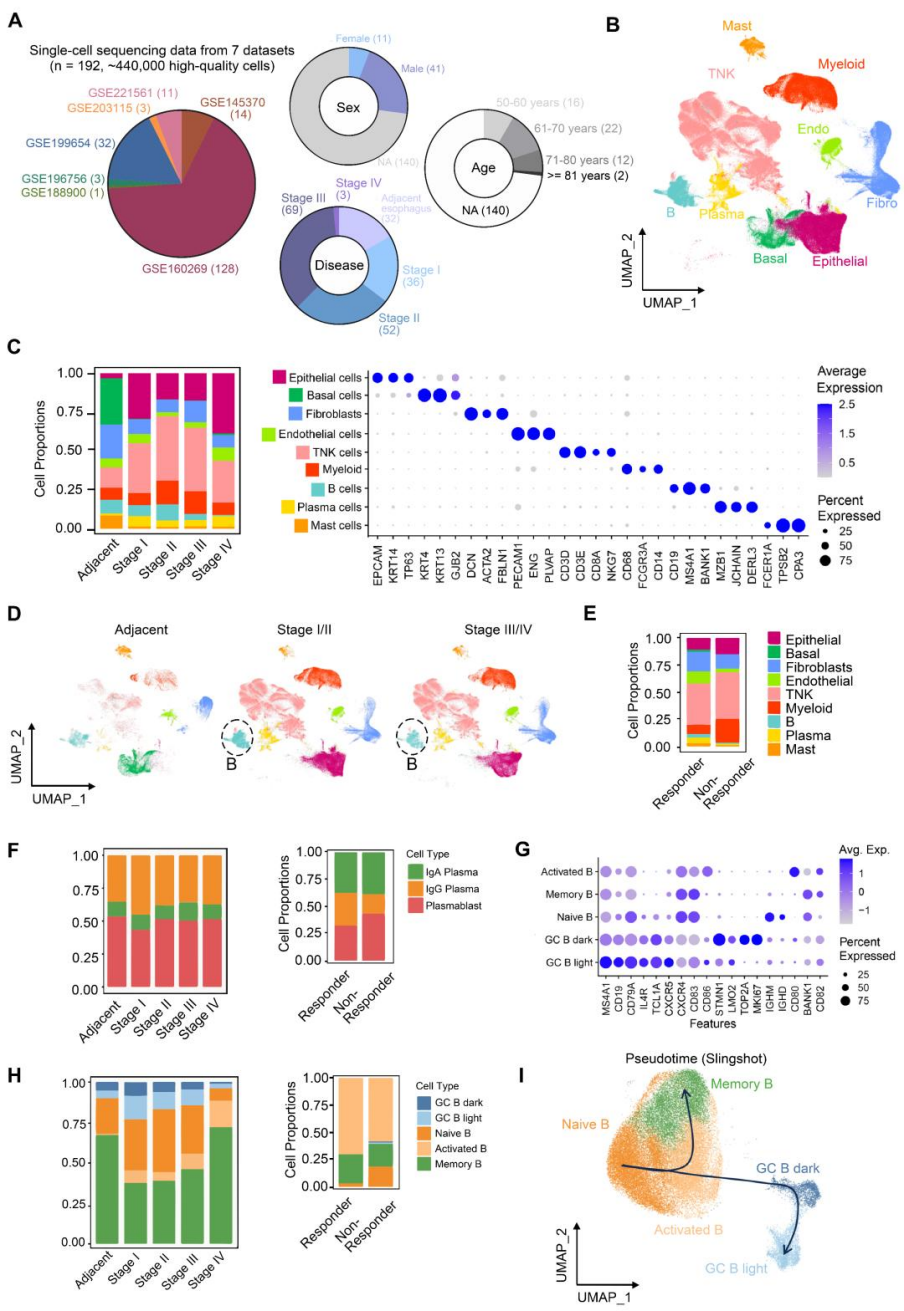
** Single-cell RNA profile of 440,000 cells of esophageal squamous cell carcinoma.** (A) Integrated datasets and patient information analyzed in this study. (B) Cell type annotations based on marker genes. (C) Proportions and gene expression profiles of cell types at different stages of ESCC. (D) Uniform Manifold Approximation and Projection (UMAP) of cell clustering, split by patient stages. (E) Proportions of cell types in patients who resistant to immunotherapy (n = 1, stage III) versus patients who respond to immunotherapy (n = 2, stage III). (F) Proportions of plasma cells in patient groups classified by different stages or immunotherapy outcomes. (G) Expression of marker genes of B cell subsets. (H) Proportions of B cells in patient groups categorized by different stages or immunotherapy outcomes. (I) Re-clustering and pseudotime analysis of single cells from the B lineage.

To characterize tumor microenvironment (TME) remodeling in ESCC, we integrated seven public scRNA-seq datasets encompassing normal tissues (n = 32) and ESCC samples across stages I (n = 36), II (n = 52), III (n = 69), and IVA (n = 3) (Figure 1A). After rigorous quality control, ~440,000 high-quality cells were retained. Batch correction resolved dataset-related batch effect using FastMNN (Figure S1A-C), enabling stable clustering of nine major cell types annotated via canonical markers (Figure 1B-C, S1C). Comparative analysis revealed progressive depletion of B cells in stage III/IV versus stage I/II, alongside reduced T cells and myeloid cells in stage IV (Figure 1C-D). Strikingly, post-immunotherapy comparison (non-responder [n = 1] vs. responders [n = 2]) showed elevated T and myeloid cells but diminished B cells in non-responders (Figure 1E).

Subtype re-clustering further delineated TME heterogeneity (Figure S2, S3). ESCC TME is composed of large amounts of T/NK cells, which was supported by independent studies 10, 11. Stage I tumors were enriched in cytotoxic CD8^+^ T cells (Figure S2A), whereas stages II/III exhibited higher exhausted CD8^+^ T cells and immunosuppressive regulatory T cells (Tregs) (Figure S2A), coupled with reduced cytotoxic CD8^+^ T cells (Figure S2A) and IgG^+^ plasma cells (Figure 1F). Stage III/IV TMEs displayed elevated tumor-associated macrophages (TAMs; predominantly M2-polarized) and inflammatory cancer-associated fibroblasts (CAFs) (Figure S2B-C), alongside increased vascular CAFs, pericytes, and microvascular endothelial cells—consistent with enhanced angiogenesis in stage IV (Figure S2C-D). These findings collectively suggest progressive immunosuppression during ESCC advancement.

Notably, despite increased activated B cells in stage III/IV (Figure 1F), plasma cell frequencies remained unchanged, indicating impaired B cell terminal differentiation. In the non-responder, germinal center (GC) B cell activation (Figure 1G-H) paradoxically coexisted with reduced IgG^+^ plasma cells (Figure 1F). Activated B cells exhibit upregulated expression of key activation and co-stimulatory molecules, including CD80, CD86, and TNFRSF13B (TACI, Figure S4A), indicating a functionally activated phenotype 27. Notably, many cells within this population show elevated expression of VIM, members of the S100A family, and structural scaffold proteins (e.g., LMNA and AHNAK), suggesting that these cells are in a transitional migratory state (Figure S4A). At this stage, they may have exited the cell cycle (Ki67⁻), while acquired enhanced tissue infiltration capacity and relied on microenvironment-derived survival signals (e.g., TNFRSF13B, LGALS1, CD82, Figure S4A). Furthermore, the upregulation of LGALS1 and KYNU suggests a role in modulating local immune responses within inflammatory niches, potentially preventing excessive immune activation. The intermediate expression of CXCR4 (Figure 1G) and a sparse BCL6 expression of BCL6 further indicate that these cells are likely to correspond to a pre-germinal center (pre-GC) B cell state (Figure S4A) 28, which is also supported by our pseudotime trajectory analysis (Figure 1I). Thus, these results supported a model wherein GC reaction dysfunction—potentially due to disrupted affinity maturation or differentiation—compromises B cell-mediated anti-tumor immunity in both ESCC progression and immunotherapy resistance.

### Cholesterol biosynthesis-related tumor cells are associated with ESCC progression and immunotherapy resistance

The immune system plays a critical role in eliminating cancer cells, yet identifying tumor cell subsets that evade immune surveillance remains pivotal for understanding immunotherapy resistance. Recent scRNA-seq study 7 have underscored tumor cell heterogeneity in ESCC, providing insights into epithelial subset dynamics and molecular mechanisms underlying progression and treatment failure.

**Figure 2:**
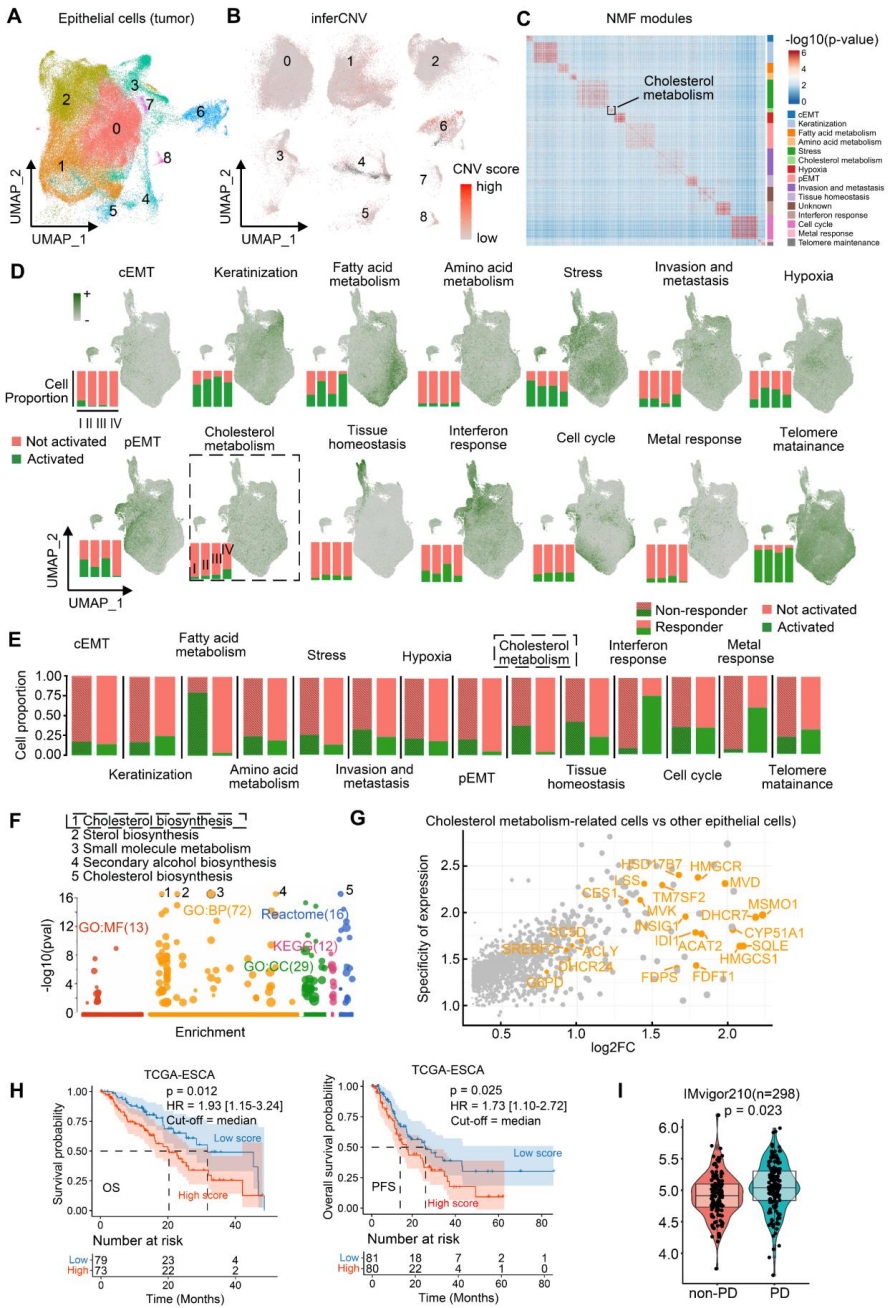
** Functional modules of epithelial malignant cells in ESCC.** (A) Re-clustering of epithelial cell subsets in ESCC. (B) InferCNV analysis identifies malignant cells in epithelial cell populations. (C) Identifying consensus functional modules in malignant cells using the Non-negative Matrix Factorization (NMF) algorithm. (D) Functional module scores and proportions of cells with activated functional modules in different stages of patients. (E) Proportion of cells with distinct activated modules between immunotherapy-resistant and responding patients. (F) Functional enrichment of module genes in cholesterol metabolism. (G) Log_2_-transformed fold change (log_2_FC) and expression specificity of genes. Differential gene expression was analyzed using Seurat FindMarkers. Gene specificity was defined as the ratio of the percentage of cells expressing a given gene in cholesterol metabolism-related tumor cells to that in other malignant cells. (H) Kaplan-Meier survival curves of ESCC patients (95% confidence interval) grouped by signature scores of cholesterol synthesis (Log-rank test). The median score was used as the cutoff. HR hazard ratio. OS/PFS overall/progression-free survival. (I) Module signature scores of cholesterol synthesis in progressive disease (PD, n = 167) or non-PD (n = 131) patients of anti-PD-L1 immunotherapy based on the IMvigor210 cohort (Mann-Whitney U test, two-tailed). Box: 25%-75% percentiles with median.

We isolated epithelial cells (n = 145,000) and employed inferCNV analysis to identify malignant populations (Figure 2A-B). In tumor tissues, epithelial cells with high CNV burden are frequently dispersed across multiple clusters defined by Seurat, rather than being confined to a single cluster, consistent with prior literature 26, 29. Subpopulations defined by principal components analysis (Seurat clusters) failed to adequately represent functional features, which prompted our use of Non-negative Matrix Factorization (NMF) to identify functionally distinct malignant modules 22. Applying NMF to 82,191 malignant cells (Figure S4B), we resolved 14 consensus functional modules, including complete EMT (cEMT), keratinization, fatty acid/amino acid metabolism, stress response, cholesterol metabolism, hypoxia, partial EMT (pEMT), invasion/metastasis, tissue homeostasis, interferon response, cell cycle, metal response, and telomere maintenance (Figure 2C). Notably, cholesterol metabolism-enriched malignant cells persisted across ESCC progression (Figure 2D) and were significantly enriched in post-treatment non-responders (Figure 2E).

Cholesterol metabolic reprogramming— spanning uptake, export, storage, and *de novo* biosynthesis—was further dissected. Key cholesterol biosynthesis genes were overexpressed in high cholesterol-metabolism cells (Figures 2F-G, Table S4). The gene set associated with cholesterol biosynthesis comprises the following key components: *MSMO1*, *DHCR7*, *MVD*, *INSIG1*, *IDI1*, *CYP51A1*, *HMGCR*, *HMGCS1*, *HSD17B7*, *TM7SF2*, *FDFT1*, *FDPS*, *CES1*, *MVK*, *SC5D*, *LSS*, *DHCR24*, *G6PD*, *SREBF2*, *ACLY*, *SQLE*, and *ACAT2*. Transcriptomic signatures of these cells (Table S5) correlated with poorer clinical outcomes. In TCGA-ESCC (n = 152), high cholesterol biosynthesis scores predicted reduced overall survival (OS) and progression-free survival (PFS) (Figure 2H).

Indeed, individual cholesterol biosynthesis genes are linked to ESCC survival. For example, high HSD17B7 expression was associated with poorer OS and PFS (Figure S4C). These findings suggest cholesterol biosynthesis-related tumor cells are closely associated with ESCC progression. IMvigor210 cohort analysis (urothelial cancer, n = 298) revealed elevated scores of cholesterol biosynthesis in immunotherapy-refractory patients (progressive disease [PD] n = 167 vs. non-PD n = 131; Figure 2I), implying that cholesterol biosynthesis-related tumor cells may correlated with cancer immunotherapy resistance. However, since the IMvigor210 cohort (anti-PD-L1) is derived from urothelial carcinoma, we further validated this hypothesis in an ESCC context by performing spatial transcriptomics and experimental validation using clinically annotated tissues from a real-world cohort of ESCC patients underwent anti-PD-1 immunotherapy.

### Spatial RNA-seq of two patients revealed cholesterol biosynthesis-related tumor cells may play a role in GC reactions

**Figure 3:**
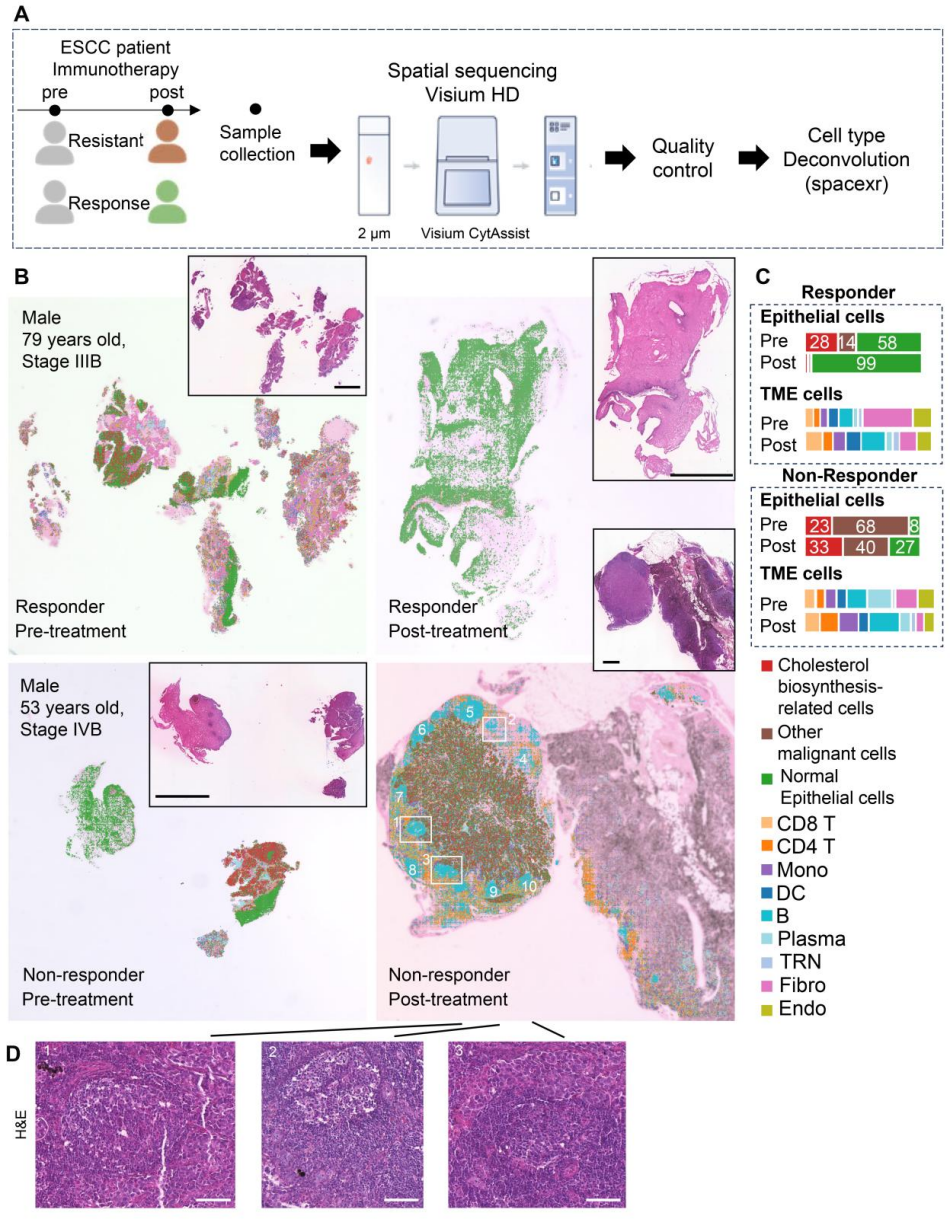
** Spatial transcriptome profile of an ESCC patient with immunotherapy.** (A) A brief overview of the experimental design for spatial analysis. The integrated single-cell dataset (~440,000 cells) with cell type annotation was used as a reference for spatial transcriptomic deconvolution. (B) Spatial cell type deconvolution, showing cell type annotations for each 8 μm × 8 μm box. The top right displays H&E data (scale bar: 1 mm) from tissue sections of the same patients used in the spatial sequencing. Based on H&E staining, normal epithelial cells exhibit an orderly arrangement, with regular nuclear morphology and lightly stained chromatin. In contrast, cancer cells display disorganized and loosely arranged architecture, pleomorphic nuclei, and prominent nucleoli, indicating markedly elevated transcriptional and metabolic activity. In post-nonresponder samples, a cloud-like zone of coagulative necrosis is observed in the right region. The tumor-stroma boundaries identified by spatial transcriptomics align well with histopathological annotations, owing to the spatially adjacent nature of the tissue sections used in the two modalities. Numbers indicate TLSs. Boxes show representative TLSs of many TLSs in the slide. (C) Proportions of cell types across different patient groups. TME: Tumor Microenvironment. (D) H&E staining of a post-nonresponder sample slide spatially adjacent to the Visium HD slide. The hallmark histological feature of a tertiary lymphoid structure (TLS) is a core of B cells (frequently exhibiting a germinal center organization) surrounded by a zone of T cells, wherein T helper cells typically outnumber T cytotoxic cells. On H&E-stained sections, the cytoplasm of these core B cells often stains pale pink, a characteristic attributed to their abundant endoplasmic reticulum and general lack of lysosomes. In contrast, the cytoplasm of the surrounding T cells stains a more basophilic blue-purple due to their higher cytoplasmic-to-nuclear ratio and greater abundance of lysosomes. While the nuclei of B cells are generally larger than those of T cells, they remain notably smaller than the enlarged, pleomorphic nuclei typically seen in carcinoma cells. Three regions correspond to those highlighted by the boxes in panel B). Scale bar: 100 μm.

To investigate spatial crosstalk between cholesterol-biosynthetic tumor cells and B lineage cells in immunotherapy outcomes, we analyzed paired pre-/post-treatment samples from two ESCC patients (n = 4 samples: 2 responder samples vs. 2 non-responder samples) using 10x Visium HD spatial transcriptomics (Figure 3A, Table S1). High-resolution spot analysis (8 μm × 8 μm) with cell type deconvolution based on our integrated single-cell reference data (Figures 3B, S5) revealed distinct TME remodeling patterns. In the responder,​ post-treatment tumors exhibited reduced cholesterol-biosynthetic tumor cells alongside increased plasma cell infiltration (Figure 3B-C). In non-responder, elevated cholesterol-biosynthetic tumor cells post-treatment coexisted with diminished plasma cells and expanded GC-enriched TLS at tumor margins (Figure 3B-D).

TLS spatial distribution was quantified via a 12-chemokine gene signature (CCL2/3/4/5/8/18/19/21, CXCL9/10/11/13; Figure S6) 5. Intriguingly, the post-treatment non-responder sample showed higher TLS scores, localized predominantly to GC regions (Figure 3B, 3D, S6). These findings highlight spatial heterogeneity within TLS/GC niches as critical determinants of immunotherapy resistance, necessitating deeper dissection of their cellular architecture.

### Cholesterol biosynthesis-related tumor cells spatially interact with GC B dark zone cells

**Figure 4:**
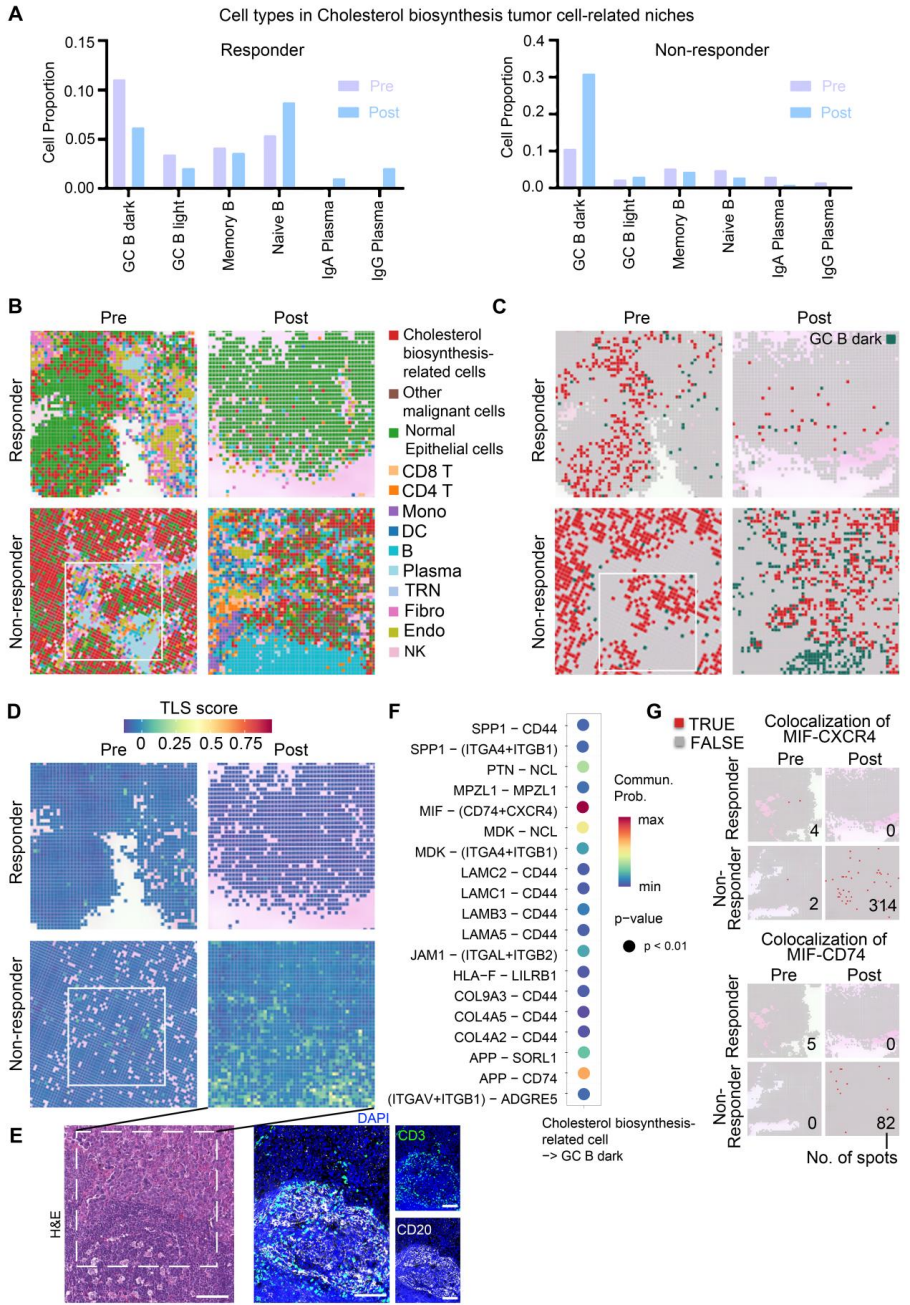
** Analysis of cholesterol biosynthesis-related niche in high-resolution spatial data.** (A) Proportion of surrounding B lineage cell types in proximity to cholesterol biosynthesis-related malignant cells. (B) Spatial feature plot (8 × 8 μm) highlighting representative regions of interest, with annotations of all major cell types. (C) Spatial feature plot (8 × 8 μm) showing representative regions of interest, focusing on cholesterol biosynthesis-related cells and GC B dark zone cells. (D) Tertiary lymphoid structures (TLS) scores (Seurat AddModuleScore) in spatial slides of patients with immunotherapy (8 × 8 μm). B), C), and D) Box indicate the same region of Figure 5B pre-non-responder sample. (E) H&E and mIHC staining to confirm the GC/TLS region of the post-non-responder sample in panel d). Scale bar: 100 μm. (F) Cell-cell communication analysis performed using CellChat. (G) Spatial feature plot illustrating colocalization of the MIF-CXCR4 and MIF-CD74 ligand-receptor pairs. Numbers are the total number of spots with a colocalization in a slide.

Thus, we further examined cell communication within the cholesterol biosynthesis-related niche by focusing on neighboring cell spots surrounding cholesterol biosynthesis-related tumor cells (Figure 4A, S7A). In the non-responder, the frequencies of GC B cells were sharply increased post-treatment within the cholesterol biosynthesis-related niche (Figure 4A). Plasma cells were successfully generated in responder tumor post-treatment, while the non-responder tumor post-treatment lacked plasma cell likely due to impaired GC reactions (Figure 4A). Spatial plots clearly displayed an increased colocalization of GC B and cholesterol biosynthesis-related tumor cells in the post-treatment sample from non-responder (Figure 4B-C). In contrast, Plasma cells in responder post-treatment have a higher tumor infiltration, whereas in non-responder post-treatment tumor, there were primarily B cells (Figure 4B). Moreover, TLS scores were higher in niches with colocalization of GC B and cholesterol biosynthesis-related tumor cells (Figure 4C-E), which suggest that cholesterol biosynthesis-related tumor cells may play a role in GC reactions.

To explore the molecular interactions between GC B cells and cholesterol biosynthesis-related cells, we performed cell-cell communication analysis using single-cell data. The MIF-CD74/CXCR4 ligand-receptor pair emerged as the most probable interaction (Figure 4F). CellChat analysis performed on the integrated single-cell dataset predicted a strong interaction probability for the MIF-(CD74+CXCR4) complex. When evaluated separately using this single-cell dataset, the MIF-CD74 interaction was predicted with a slightly higher probability (21.3%) than MIF-CXCR4 (19.8%). Although the MIF-CD74 interaction appeared high frequency, the majority of samples in the single-cell dataset lacked treatment information, and the sequencing was based on dissociated cells, potentially missing relevant spatial context. To better understand these interactions in the context of immunotherapy, we further analyzed our spatial transcriptomics data, which showed a higher degree of MIF-CXCR4 co-localization in post-treatment tumor of non-responder (Figure 4G), suggesting that this interaction may be linked to immunotherapy resistance.

Interestingly, cell-cell communication analysis also revealed that *MIF* expression was highest in cholesterol biosynthesis-related cells compared to other TME cell types (Figure S7B). It was demonstrated MIF 30 and cholesterol biosynthesis 31, as well as HMGCR 32, 33—a rate-limiting enzyme in cholesterol synthesis—are upregulated in ESCC tumor cells. Our immunohistochemistry data demonstrated high expression of both HMGCR and MIF in a subset of ESCC tumor cells (Figure S8). Analysis of both scRNA-seq (Figure S7C) and spatial transcriptomic (Figure S7D) data revealed that *MIF* is expressed across various cell types, with the highest levels observed in malignant cells, particularly those identified as cholesterol biosynthesis-related tumor cells. Consistently, the cholesterol biosynthesis signature score and the expression of *HMGCR* were also most elevated in this specific tumor cell population. Compared to single-cell data, the proportion of *MIF* and *HMGCR* expression in cholesterol-synthesizing tumor cells were lower in spatial transcriptomics. This is likely because each spot captures only a portion of a cell (at most 5 µm thick and 8 µm wide) and may contain RNAs from two adjacent cells. These findings indicate a specific association between MIF and upregulated cholesterol biosynthesis. Furthermore, although MIF expression has been reported in macrophages, these cells are found at very low frequency within GC regions (Figure S7E). Although detailed mechanism remains unclear, these results suggest a positive correlation between elevated cholesterol biosynthesis and MIF expression, which have been observed in animal models 34. In summary, these data suggested a hypothesis that cholesterol biosynthesis-related tumor cells with highly expressed *MIF* may correlate with disrupted GC reactions.

### Multiplex immunohistochemistry revealed impaired GC reactions driven by MIF^+^ tumor cells

**Figure 5:**
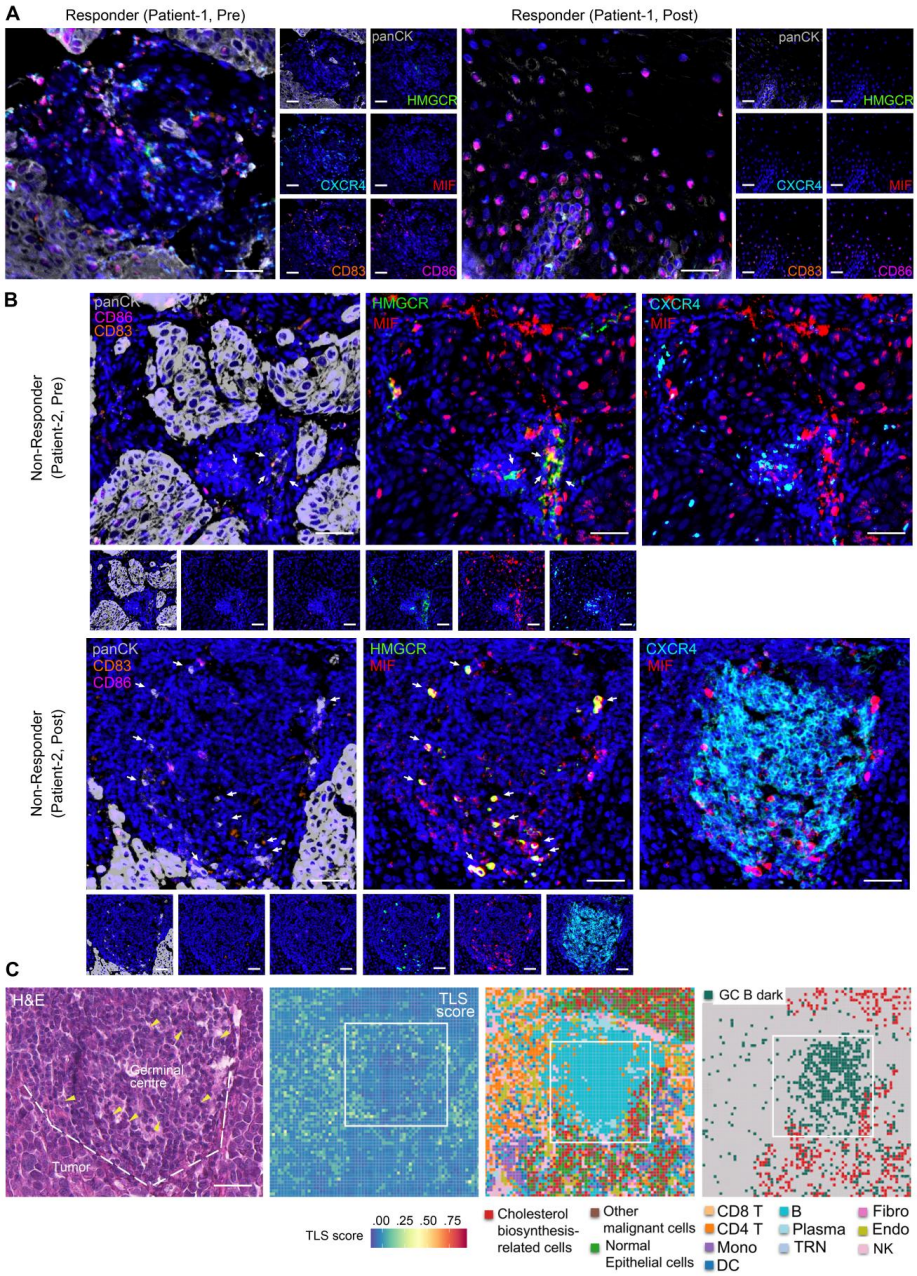
** Cholesterol biosynthesis-related tumor cells impair GC reactions**. (A, B) Representative images of multiplex immunohistochemistry (mIHC) showing the expression and colocalization of ligand MIF from cholesterol biosynthesis-related tumor cells (high HMGCR and MIF, and positive Pan-CK) and receptor CXCR4 of GC B cells (high CXCR4, low CD83 and CD86) (n = 7 patients, scale bar: 50 μm). Arrows indicate panCK^+^HMGCR^+^MIF^+^ cells. The upper slide (pre-treatment, non-responder) of B) shares spatial regions with the regions in white boxes of Figures 4B-D. (C) H&E staining and spatial transcriptome profile (8 × 8 μm) for a non-responder of post-treatment sample. Boxes indicate the same region as the mIHC of panel B). Left to right: H&E (scale bar: 50 μm), TLS scores, Cell type annotations, and Spatial distribution of cholesterol biosynthesis-related tumor cells and GC B dark zone cells. Arrows in H&E indicate nuclei with characteristic morphology of tumor cells. Dashed lines in H&E distinguish the tumor and GC/TLS regions.

**Figure 6:**
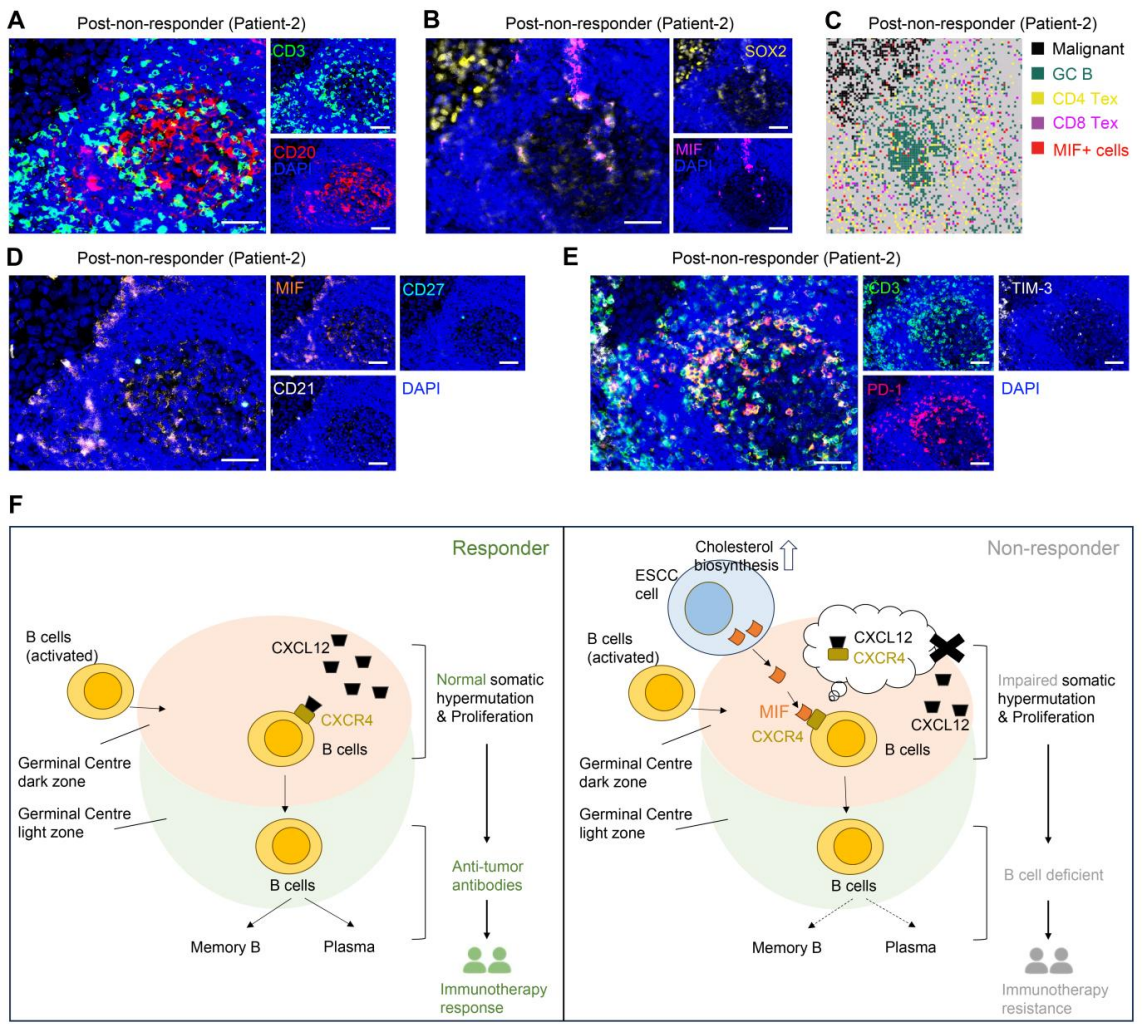
** MIF^+^ tumor cells are associated with compromised B cell immune response.** (A) Representative images of multiplex immunohistochemistry (mIHC) showing the GC/TLS regions. Scale bar: 50 μm. (B) Representative images of mIHC data showing the MIF^+^ tumor cell infiltrating in GCs. Scale bar: 50 μm. (C) Spatial data showing the exhausted T cells in MIF^+^ regions. (D) Representative images of mIHC data showing the exhausted B cells in MIF^+^ regions. Scale bar: 50 μm. (E) Representative images of mIHC data showing the exhausted T cells in GC/TLS. Scale bar: 50 μm. (F) Schematic diagram illustrating the proposed model of cholesterol biosynthesis-related tumor cells in the immune response.

Based on mIHC assay, we revealed abundant CD83^+^ and CD86^+^ antigen-presenting cells within peritumoral stroma of pre- and post-treatment samples of responders (Figure 5A, S9A-B, S9D), indicative of enhanced anti-tumor immunity. Notably, MIF-CXCR4 interactions were absent in both pre- and post-treatment of responders (Figure 5A, S9A-B, S9D). In contrast, non-responders exhibited persistent MIF-CXCR4-mediated crosstalk between GC B cells (CXCR4^hi^CD83^lo^CD86^lo^ dark-zone subset) and cholesterol-biosynthetic tumor cells (pan-CK^+^, HMGCR^hi^) across treatment timepoints (Figure 5B, S9C). H&E staining and spatial transcriptomics of the non-responder's post-treatment tumor further validated the interactions between GC B and cholesterol synthesis-related tumor cells within TLS/GC regions (Figure 5C). The same phenomenon was frequently observed in other TLS regions (Figure 3D ROI 1, Figure S10). Tumor cell infiltrating in TLS/GC region was further confirmed by mIHC data (Figure 6A-B). Strikingly, baseline pre-treatment tumors of non-responders already exhibited low-frequency MIF-CXCR4 interactions (Figure 4B-D white boxes, Figure 5B, Figure S9C), suggesting potential early GC dysfunction before therapy. We noticed that MIF is also expressed by other tumor cells in pre-nonresponders that do not express HMGCR (Figure 5B). This phenomenon supports our finding that high MIF expression is a key factor driving treatment non-response, indicating that MIF upregulation is a broader phenomenon not restricted solely to the tumor cell subpopulation expressing high HMGCR.

Consistently, spatial transcriptome and mIHC analyses using adjacent sections show that TLS regions containing MIF^+^ tumor cells (SOX2^+^) are enriched with exhausted T cells (CD3^+^PD-1^+^TIM-3^+^) and exhausted B cells (CD20^+^CD21^-^CD27^-^PD-1^+^), indicating an impaired B-cell immune response (Figure 6A, 6C-E). Mechanistically, tumor-derived MIF competitively inhibits CXCL12-CXCR4 binding—a canonical pathway essential for GC B cell affinity maturation 35—thereby impairing antibody-driven immunity (Figure 6F). This tumor cell-driven hijacking of CXCR4 signaling provides a spatial mechanism for TME reprogramming and immunotherapy resistance.

## Discussion

B cells play a crucial role in anti-tumor immunity. As a critical complement to cellular immunity, they suppress tumor progression, particularly in late-stage tumors where abundant immunosuppressive Treg cells and exhausted T cells accumulate 36. B cells migrate from peripheral immune organs to tumor sites, where antigenic stimulation activates them and initiates GC reactions 6. During this process, dark zone GC B cells undergo clonal expansion and somatic hypermutation, mediated by the CXCR4-CXCL12 signaling axis 35.

CXCR5 then directs GC B cells to the light zone, where CXCL13-mediated interactions drive positive selection of high-affinity antibody-producing B cells 35. Repeated recirculation between zones refines selection, ultimately yielding antibody-secreting B cells that participate in immune responses. While pathological GC assessment in tumors predicts immunotherapy response, some patients exhibit GC-rich tumors yet remain resistant 3, underscoring functional heterogeneity within GCs. Our data demonstrate that tumor cells disrupt GC reactions via MIF-CXCR4 interactions, suggesting that GC evaluation, combined with MIF or HMGCR assessment, could refine biomarkers for immunotherapy sensitivity. Thus, beyond GC presence, evaluating MIF or HMGCR expression is critical to determine GC functional integrity and optimize therapeutic strategies.

MIF (macrophage migration inhibitory factor) is a multifunctional proinflammatory cytokine that promotes the secretion of cytokines such as TNF and IL-2/6/8 37. Beyond its role in innate immunity, MIF exerts diverse functions through engagement with multiple receptors, leading to context-dependent outcomes in both physiological and pathological settings. A key mechanism of MIF activity involves its interaction with various chemokine receptors. MIF can bind directly to CXCR2 and CXCR4, inducing monocyte chemotaxis 38. Furthermore, it facilitates the formation of CD74/CXCR2 and CD74/CXCR4 complexes, which enhance monocyte retention via upregulation of adhesion molecules. Notably, MIF functions as a non-canonical ligand for CXC-family receptors: it competes with CXCL8 for binding to CXCR2 and with CXCL12 for CXCR4, thereby modulating immune cell recruitment 38. This competitive binding highlights MIF's capacity to disrupt conventional chemokine signaling pathways. The functional pleiotropy of MIF arises from its ability to engage both cognate (e.g., CD74) and non-cognate (e.g., CXCR2, CXCR4, CXCR7) receptors, forming distinct signaling complexes that elicit cell- and microenvironment-specific responses 38. Importantly, this mechanism supports our hypothesis: MIF abundantly secreted by tumor cells may disrupt the CXCR4-CXCL12 axis, interfering with germinal center reactions and thereby contributing to immune evasion.

Cholesterol is a crucial structural component of the plasma membrane, and its metabolism involves several processes, including biosynthesis, uptake, export, and esterification 39. Cholesterol biosynthesis primarily occurs in hepatocytes, with other cells producing smaller amounts under normal physiological conditions. However, in tumors, cholesterol often accumulates and contributes to tumor growth 40. Our study observed that specific ESCC cells exhibit significant upregulation of genes associated with cholesterol biosynthesis. Among these, *HMGCR*, a core rate-limiting enzyme in cholesterol biosynthesis and a target of statins 41, was found to be one of the most differentially expressed genes. Based on this, we categorize this subset of cells as being characterized by enhanced cholesterol biosynthesis.

It is important to note that cholesterol metabolism is critical in regulating anti-tumor immune responses by influencing various immune cells involved in innate and adaptive immunity. Previous studies have shown that elevated cholesterol levels can regulate macrophage polarization 42, enhance T cell proliferation and differentiation 43, inhibit NK cell release of IFN-γ 44, and promote IL-10 release by regulatory B cells 45. For instance, cholesterol can induce monocyte expansion and cholesterol ester accumulation, triggering inflammasome and activating NLRP3, leading to cell death and releasing pro-inflammatory cytokines such as IL-1β and IL-18 46. Interestingly, an atherosclerosis animal model study 34 demonstrated that cholesterol feeding significantly increased MIF levels in New Zealand white rabbits, whereas those fed a normal diet did not exhibit elevated MIF levels. This suggests that elevated cholesterol induces MIF upregulation, which aligns with our own findings in ESCC cells. Specifically, ESCC cells exhibiting enhanced cholesterol biosynthesis also show the highest levels of MIF, although the exact molecular mechanism remains unclear. These observations suggest that abnormal accumulation of cholesterol in ESCC cells was associated with local MIF elevation in GCs, which disrupts GC reactions and impairs B cell immunity through MIF-CXCR4 interaction, contributing to immunotherapy resistance. These findings highlight potential for combining immune therapy with MIF inhibitors or statin treatment in the future, although the feasibility of this approach requires further validation through animal models and prospective clinical studies.

It is crucial to acknowledge the limitations of this study. In single-cell data, we observed elevated T and myeloid cells but diminished B cells post-immunotherapy in one non-responder compared to two responders. Although we validated this phenomenon in our longitude comparison of pre- and post-treatment samples from the same patients, sample size is limited. Second, our Visium HD analysis was based on paired pre- and post-treatment samples from only two patients. Although a total of seven patients were included in the experimental validation, obtaining post-treatment samples remains challenging. Moreover, a caveat is that the IMvigor data were derived from urothelial carcinoma, not ESCC. While cholesterol metabolic dysregulation appears in multiple malignancies, tumor-type-specific factors (e.g., immune microenvironment, driver mutations) may modulate its functional impact. Finally, all pre-treatment samples from the seven patients included in this study were collected within the two-week period preceding treatment initiation. Given the potential influence of sampling timing on the results, future studies with larger, prospectively designed longitudinal cohorts are warranted to further validate these observations and to systematically evaluate the impact of temporal variations.

## Conclusion

Initial analysis of scRNA-seq datasets from two immunotherapy responders and one non-responder revealed a paradoxical increase in GC B cells within non-responder tumors, challenging conventional views of B cell-mediated anti-tumor immunity. This observation prompted mechanistic investigation into GC dysfunction in ESCC immunotherapy resistance. Through high-resolution spatial analysis of paired pre-/post-treatment tumors, we demonstrated that although GC reactions were activated in non-responders, MIF-expressing tumor cells disrupted canonical CXCL12-CXCR4 signaling via direct MIF-CXCR4 interactions. This competitive disruption impaired affinity maturation of GC B cells, suppressed plasma cell differentiation, and ultimately compromised B cell-mediated anti-tumor immunity, driving therapeutic resistance. While TLS presence is often associated with favorable immunotherapy outcomes, our multi-omics data and mIHC validation challenge this paradigm by revealing functional TLS heterogeneity. We propose that MIF^+^ tumor cell infiltration within TLS, rather than TLS abundance alone, is a critical co-factor for predicting immunotherapy response. Future studies in larger cohorts are warranted to validate this mechanism and explore therapeutic strategies targeting the MIF-CXCR4 axis to restore GC functionality.

## Supplementary Material

Supplementary figures. **Figure S1**. UMAP of scRNA-seq data of 440,000 cells. **Figure S2**. Re-clustering analysis of 4 major cell types. **Figure S3**. Subtypes marker expression and proportions in immunotherapy responder or non-responder. **Figure S4**. Single-cell analysis of B cells and malignant cells. **Figure S5**. Inferred copy number variation (CNV) profile of spatial transcriptome data. **Figure S6**. Tertiary lymphoid structures (TLS) scores in spatial slides of patients with immunotherapy. **Figure S7**. Cell proportions in responder and non-responder to immunotherapy. **Figure S8**. Immunohistochemistry analysis of HMGCR and MIF protein expression using ESCC tissues. **Figure S9**. mIHC analysis of MIF-CXCR4 interactions between cholesterol biosynthesis-related tumor cells and GC B cells. **Figure S10**. Representative images of multiplex immunohistochemistry (mIHC) in the non-responder post-treatment sample.

Supplementary tables. **Table S1**. Patient characteristics and sample information of spatial transcriptome sequencing. **Table S2**. Patient characteristics, sample information, and cell quality/filtering of single-cell RNA-seq data. **Table S3**. Cell markers used for cell type annotation in single-cell RNA-seq analysis. **Table S4**. Functional enrichment results based on module genes of cholesterol metabolism-related genes. **Table S5**. Marker genes of cholesterol metabolism-related tumor cells compared to other malignant cells.

## Abbreviations

ESCC: esophageal squamous cell carcinoma
scRNA-seq: single-cell RNA sequencing
TME: tumor microenvironment
mIHC: multiplex immunohistochemistry
TLS: tertiary lymphoid structures
GC: germinal center
FFPE: Formalin-Fixed and Paraffin-Embedded
RCTD: Robust Cell Type Decomposition
RECIST: Response Evaluation Criteria in Solid Tumors
EMT: epithelial-mesenchymal transition
MIF: macrophage migration inhibitory factor
PR: partial response
PD: progressive disease
H&E: hematoxylin and eosin staining
NMF: Non-negative Matrix Factorization
