# Supplementary material for: MIF-expressing tumor cells mediate immunotherapeutic resistance in esophageal squamous cell carcinoma
Source: Theranostics. 2026 Jan 1;16(3):1613–29. doi: 10.7150/thno.118269 (PMC12679699; doi:10.7150/thno.118269)
Supplement: Supplementary file 1 — Supplementary figures. Figure S1. UMAP of scRNA-seq data of 440,000 cells. Figure S2. Re-clustering analysis of 4 major cell types. Figure S3. Subtypes marker expression and proportions in immunotherapy responder or non-responder. Figure S4. Single-cell analysis of B cells and malignant cells. Figure S5. Inferred copy number variation (CNV) profile of spatial transcriptome data. Figure S6. Tertiary lymphoid structures (TLS) scores in spatial slides of patients with immunotherapy. Figure S7. Cell proportions in responder and non-responder to immunotherapy. Figure S8. Immunohistochemistry analysis of HMGCR and MIF protein expression using ESCC tissues. Figure S9. mIHC analysis of MIF-CXCR4 interactions between cholesterol biosynthesis-related tumor cells and GC B cells. Figure S10. Representative images of multiplex immunohistochemistry (mIHC) in the non-responder post-treatment sample. [file thnov16p1613s1.pdf]

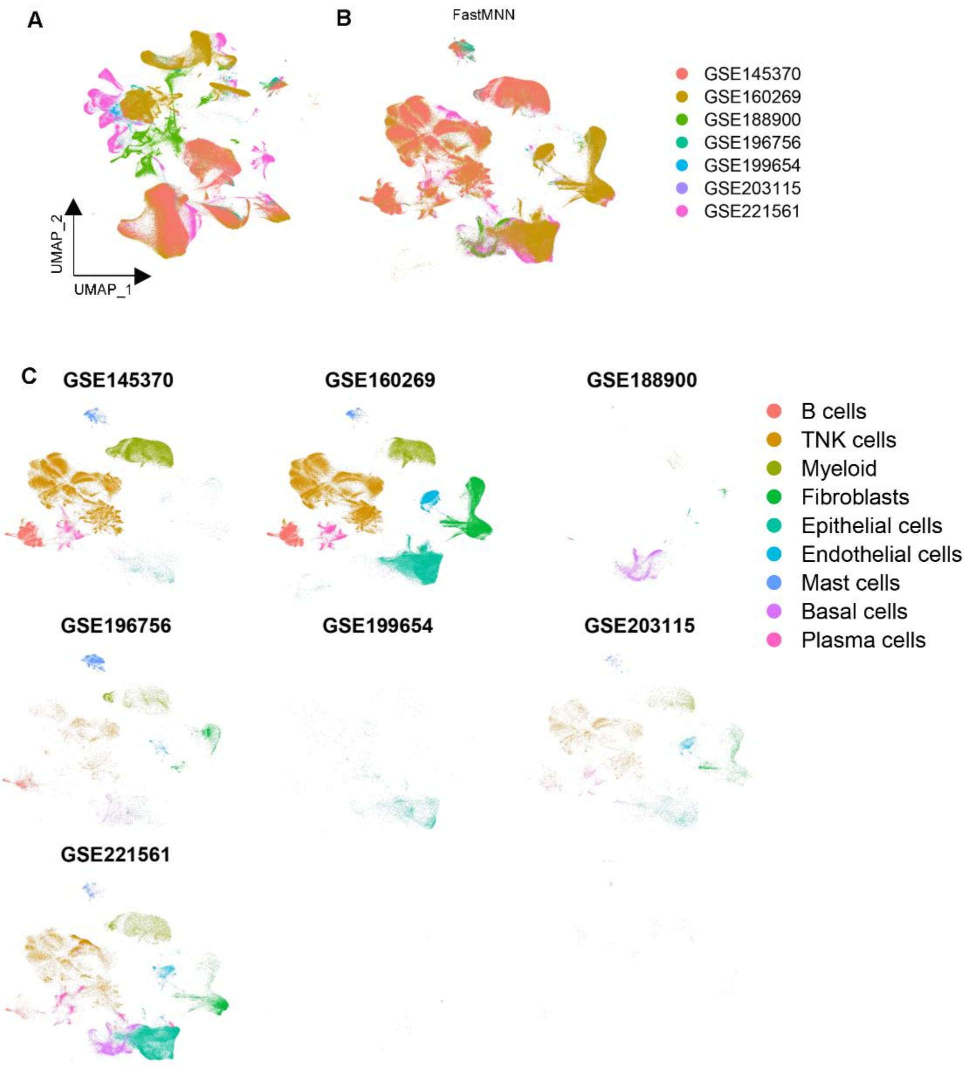

Figure S1. **UMAP of scRNA-seq data of 440,000 cells**. A, B) UMAP of the single-cell clustering: A) without batch removal and B) with batch removal. C) The UMAP of cell clustering split by different datasets. For GSE188900 and GSE196756 datasets, only the normal esophagus samples were included; thus, the UMAP of these two datasets show primarily basal/epithelial cells and have low immune cell infiltrating. GSE145370 lack of tumor cells because originally the cells of this dataset were $CD45^{+}$ sorted. The numbers of cells in each sample before and after filtering are provided in Supplementary table S2.

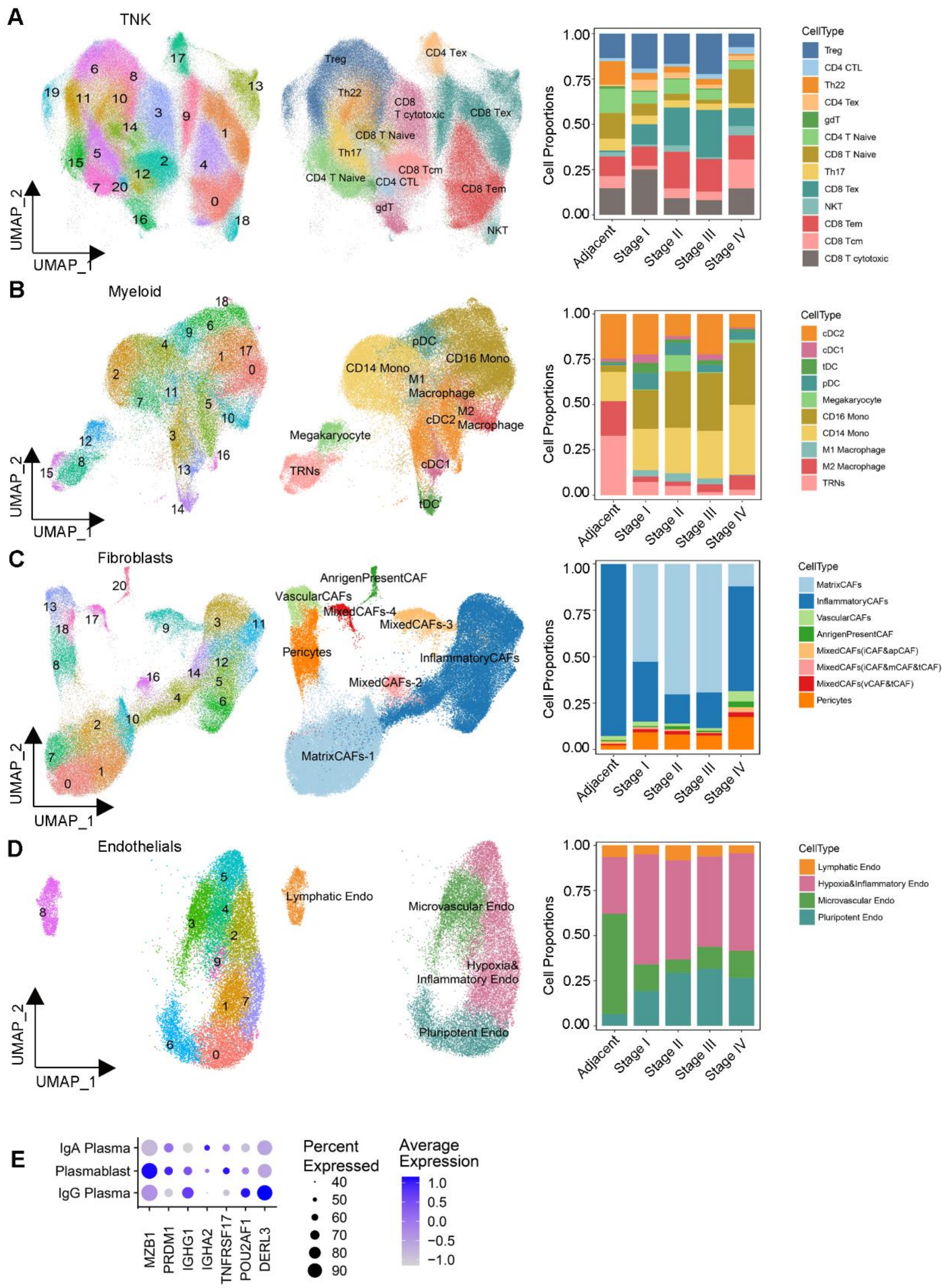

Figure S2. **Re-clustering analysis of 4 major cell types.** A) TNK, B) myeloid, C) fibroblasts, and D) endothelial cells in different stages. E) Marker expression of plasma cell subsets.

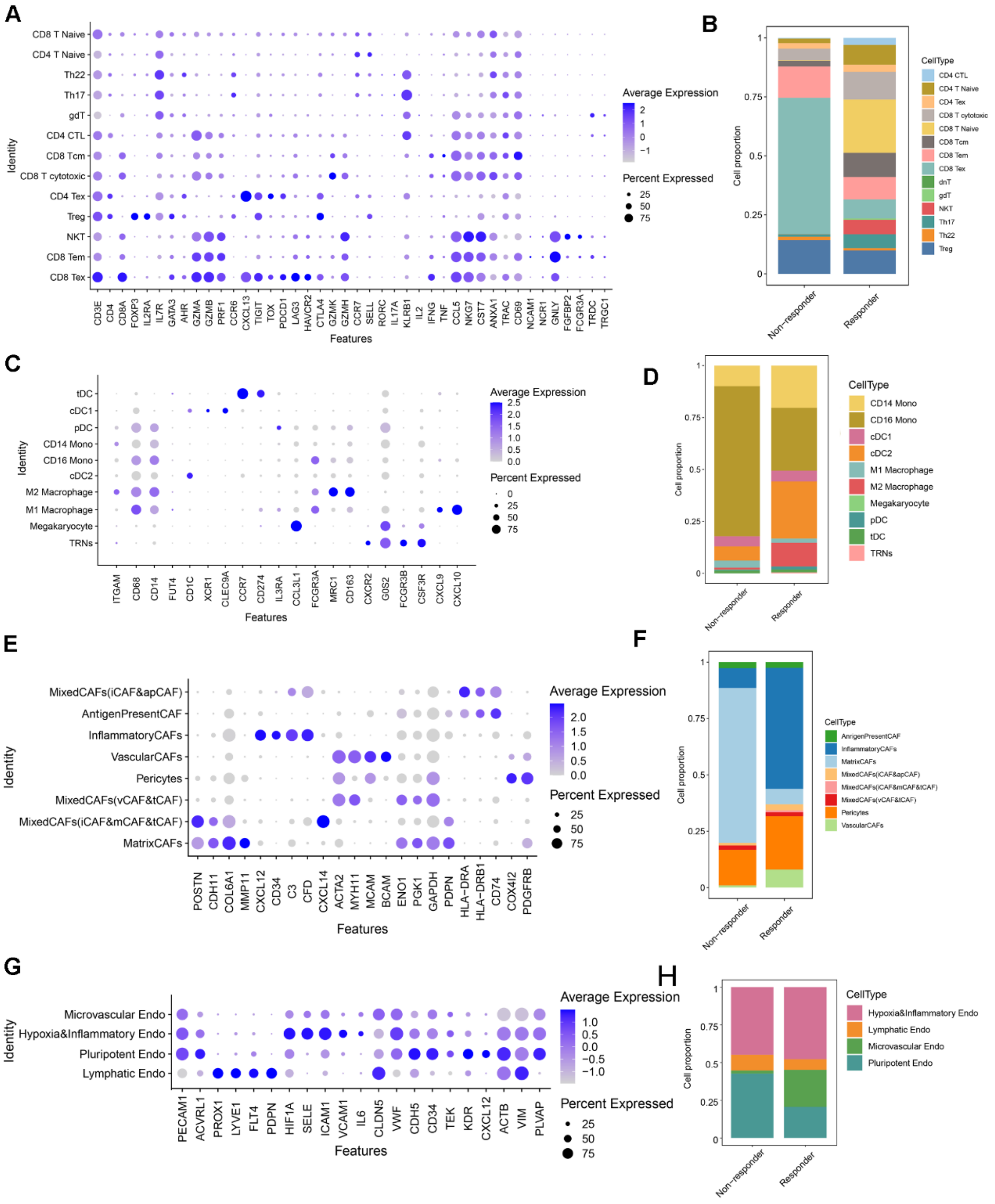

Figure S3. **Subtypes marker expression and proportions in immunotherapy responder or non-responder**. A, C, E, G) The expression of marker genes expression in all scRNA-seq samples and B, D, F, H) the proportions of cell subsets in responders (n = 2) and non-responder (n = 1).

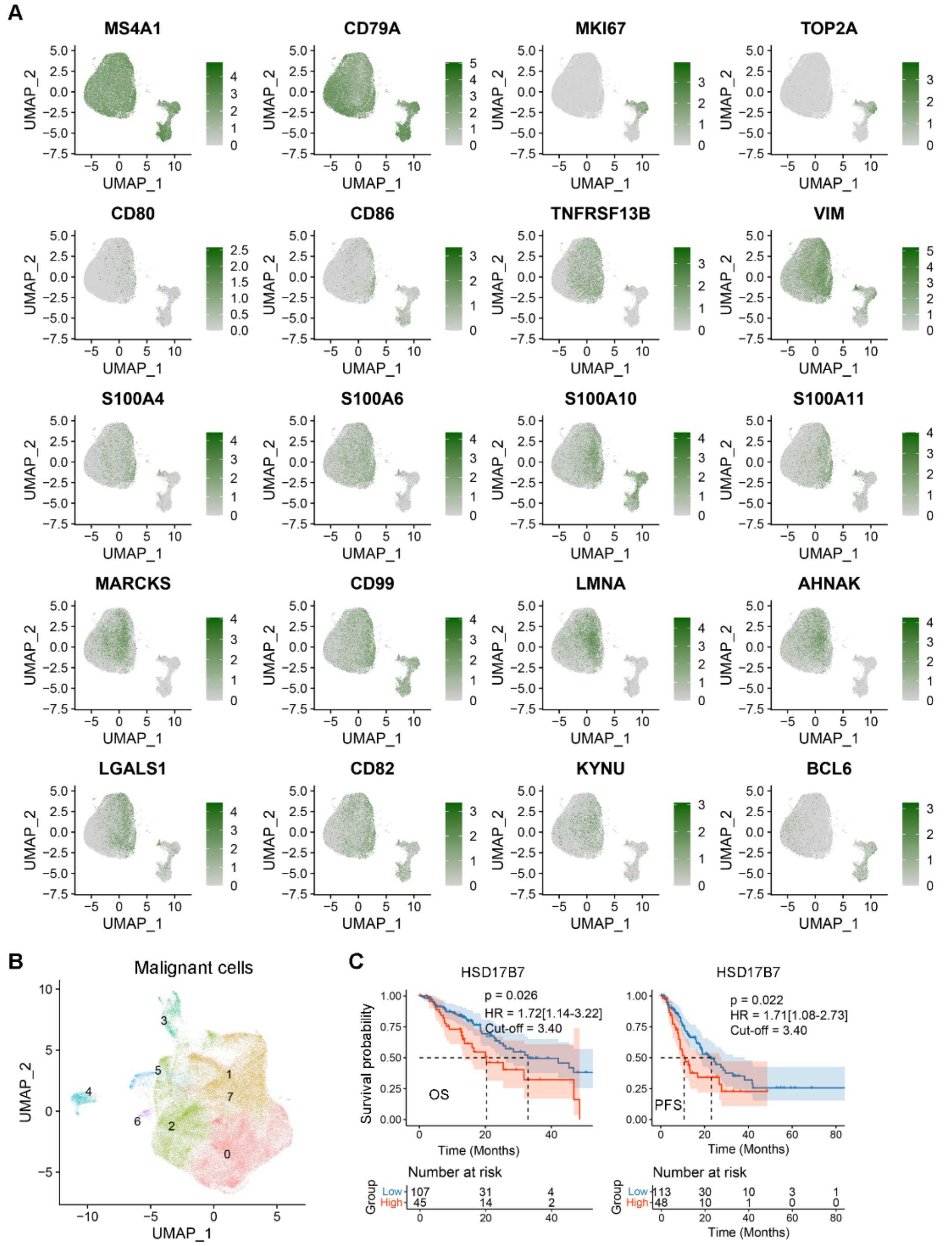

Figure S4. Single-cell analysis of B cells and malignant cells.

A) UMAP of B cells for the gene expression of activated B cell functions. B) UMAP of malignant cells and clustering based on Seurat. C) Kaplan-Meier curves showing the patient survival between high- and low-score of cholesterol biosynthesis signature using the TCGA-ESCA dataset.

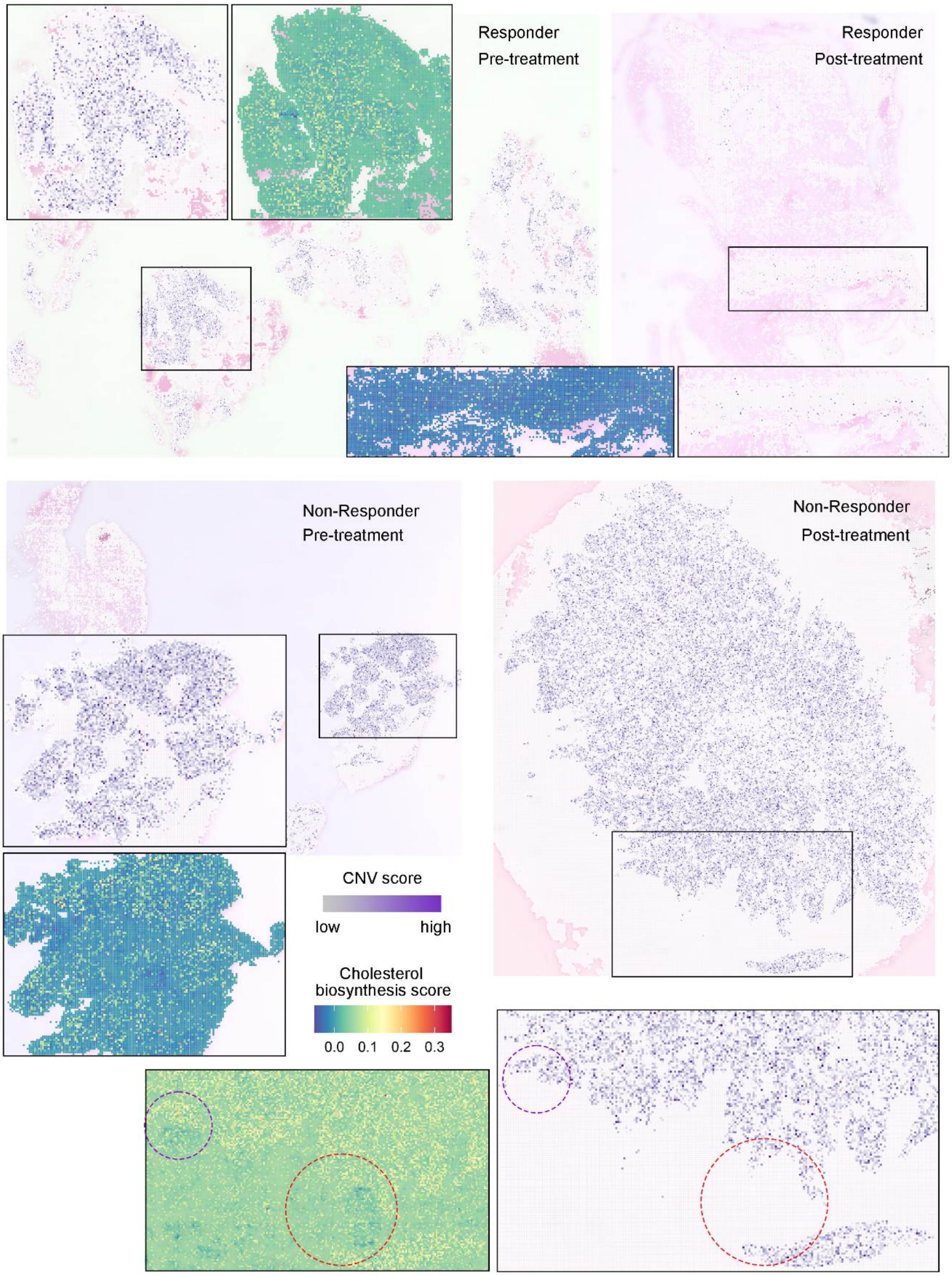

Figure S5. **Inferred copy number variation (CNV) profile of spatial transcriptome data.** Boxes showing the CNV scores and cholesterol biosynthesis signature scores. Purple circles indicate the same area as in Figure 4B-D. Red circles indicate the same area as in Figure 5C, which has been flipped vertically to align with the orientation of the mIHC panels.

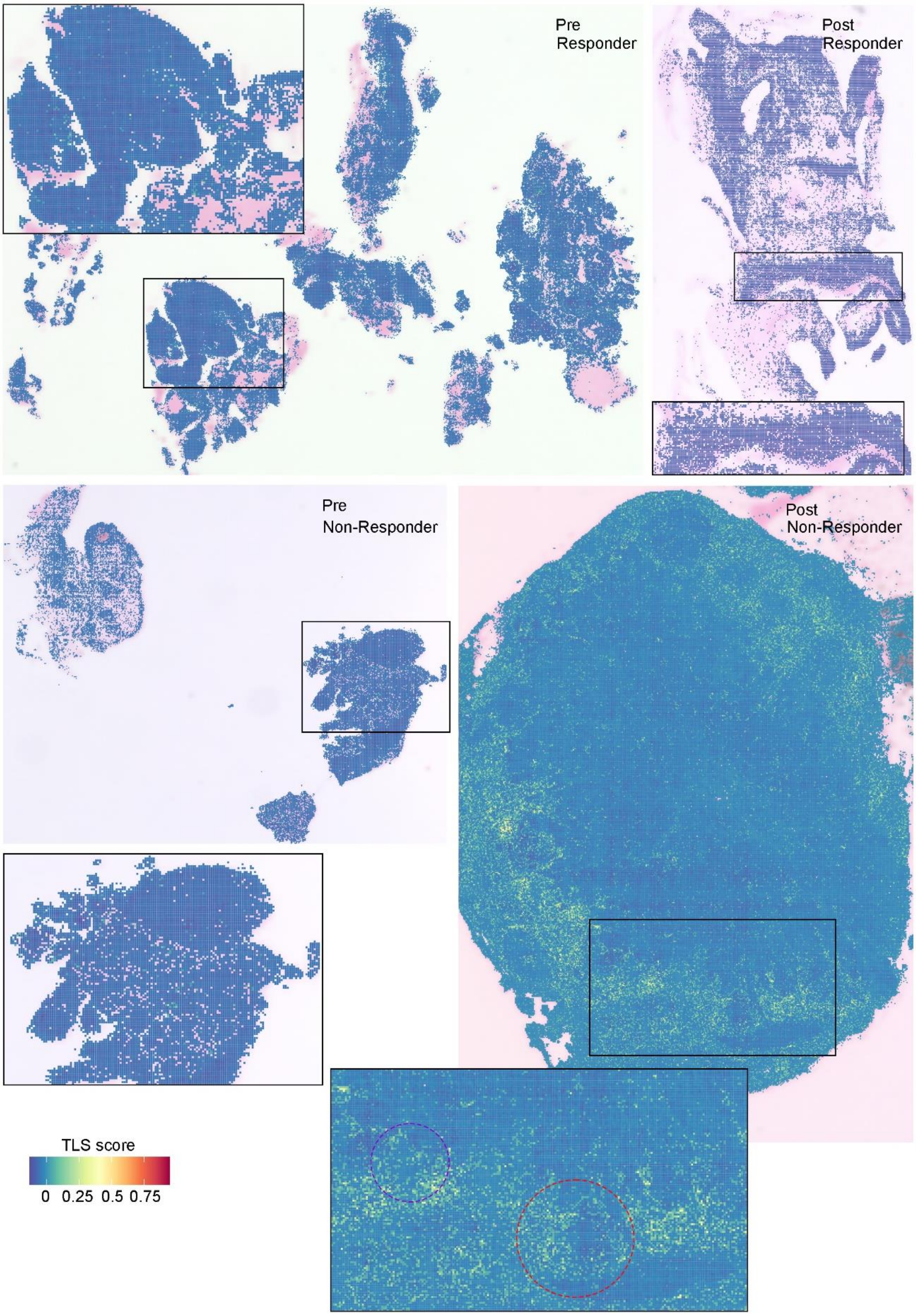

Figure S6. **Tertiary lymphoid structures (TLS) scores in spatial slides of patients with immunotherapy.** Boxes: Room-in regions. Purple circle indicates the same area as in Figure 4B-D. Red circle indicates the same area as in Figure 5C, which has been flipped vertically to align with the orientation of the mIHC panels.

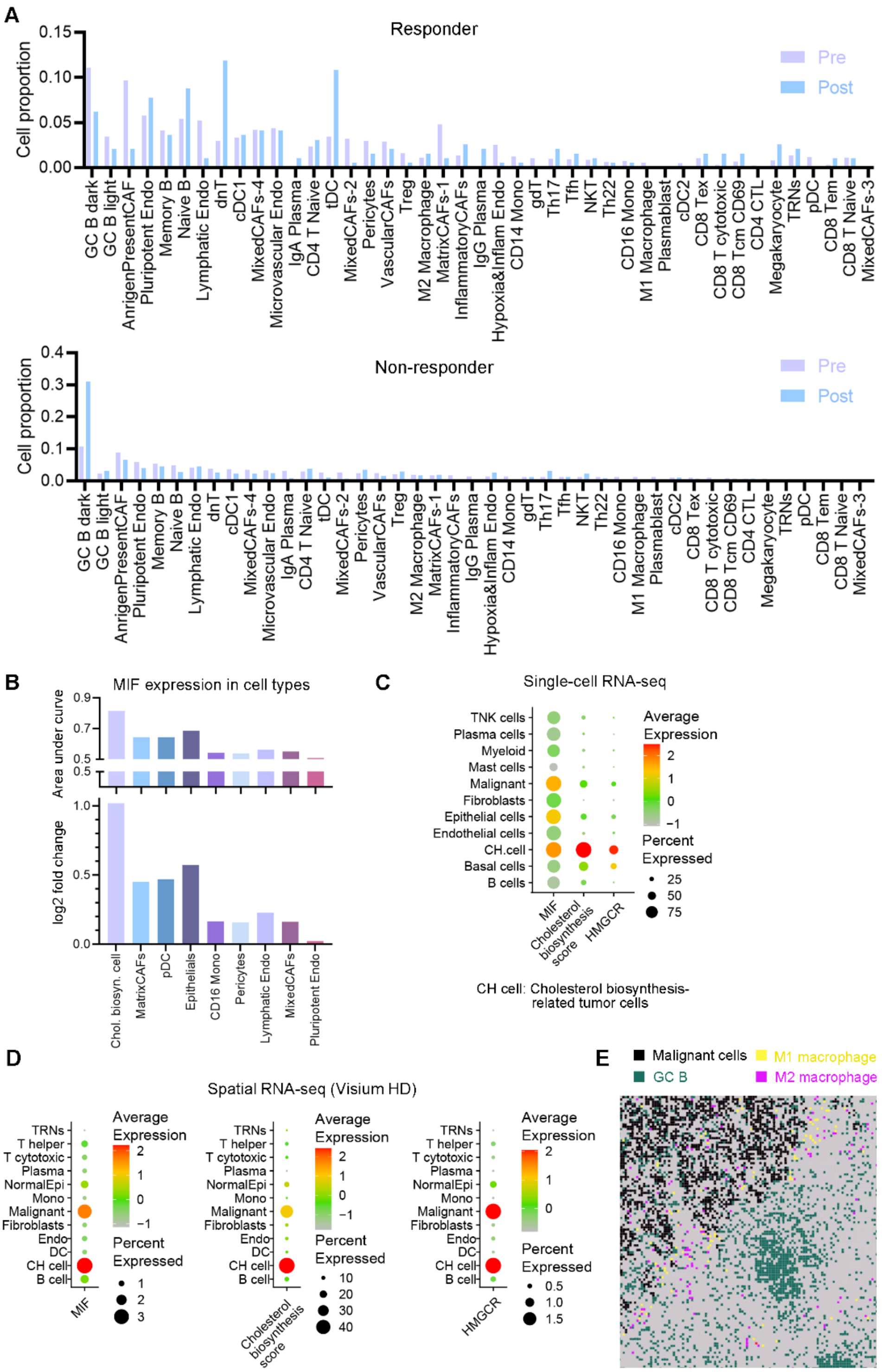

Figure S7. **Cell proportions in responder and non-responder to immunotherapy.** A) Proportion of surrounding cell types in proximity to cholesterol biosynthesis-related malignant cells. B) Statistical analysis of MIF expression across cell types by CellChat. C) and D) Cholesterol synthesis signature score (AddModuleScore) and expression of MIF/HMGCR in cell types in single-cell and spatial data. E) Spatial distribution of macrophages and GC B cells in a post-treatment sample of non-responder (Patient-2).

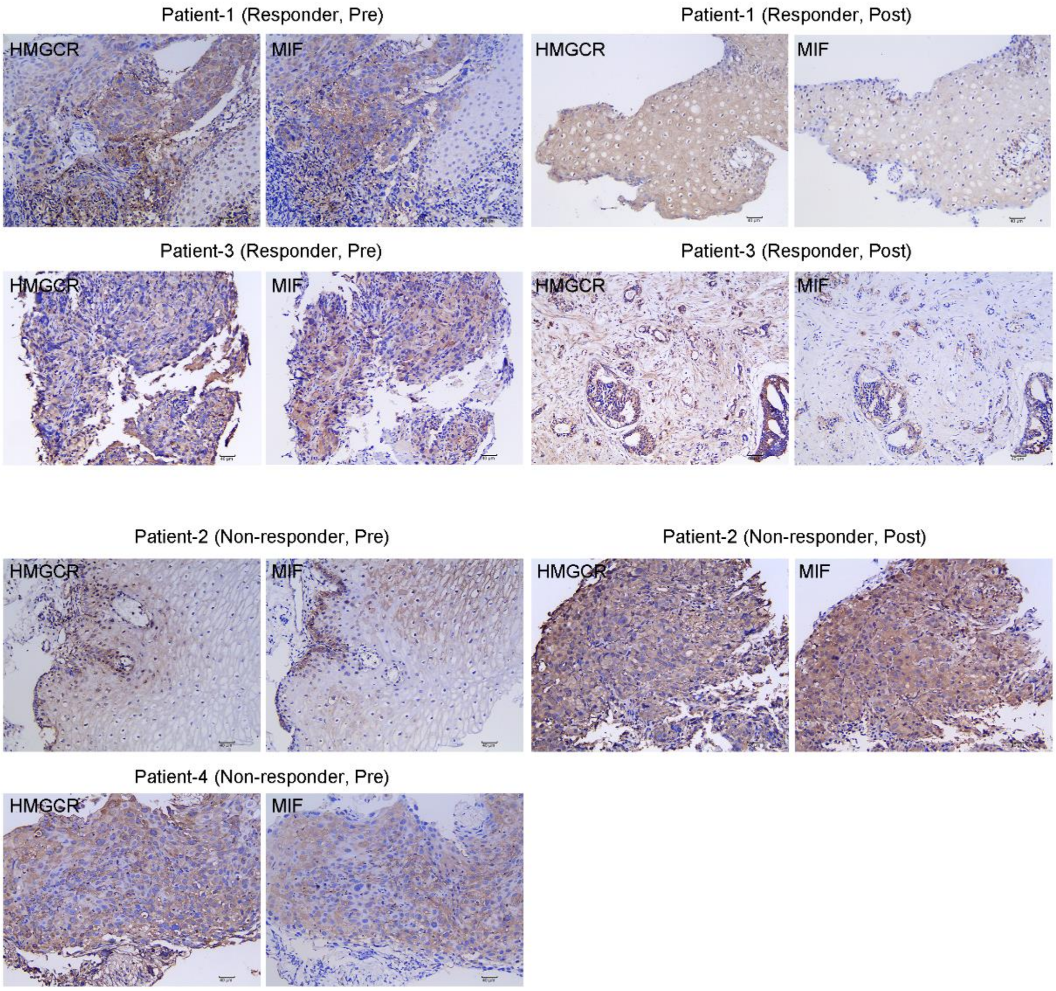

Figure S8. Immunohistochemistry analysis of HMGCR and MIF protein expression using ESCC tissues. Scale bar: 40 μm. Immunohistochemistry analysis revealed co-expression of HMGCR and MIF in a subset of tumor cells in ESCC tissues, both before and after treatment. Primary antibodies against HMGCR (Huabio, ET1702-41, 1:150 dilution) and MIF (Cell Signaling Technology, 87501, 1:150 dilution) were used.

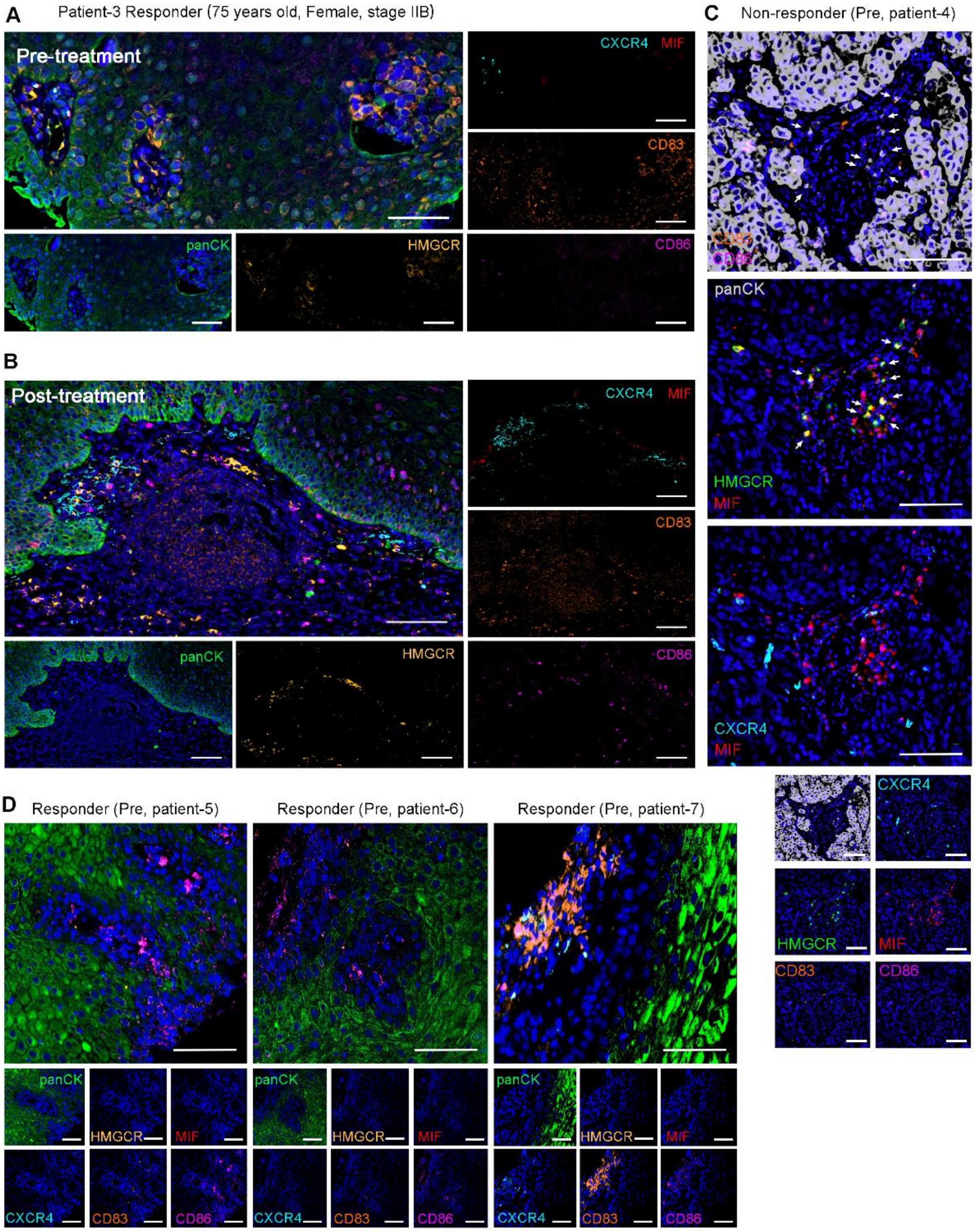

Figure S9. **mIHC analysis of MIF-CXCR4 interactions between cholesterol biosynthesis-related tumor cells and GC B cells**. A) pre- and B) post-treatment samples from a female patient who underwent immunotherapy were shown. C,D) mIHC analysis of pre-treatment samples from four additional patients showing MIF-CXCR4 interactions were observed in the C) non-responder rather than in D) responders. Scale bar: 100 μm. Arrows in C) indicate $panCK^{+}HMGCR^{+}MIF^{+}$ cells.

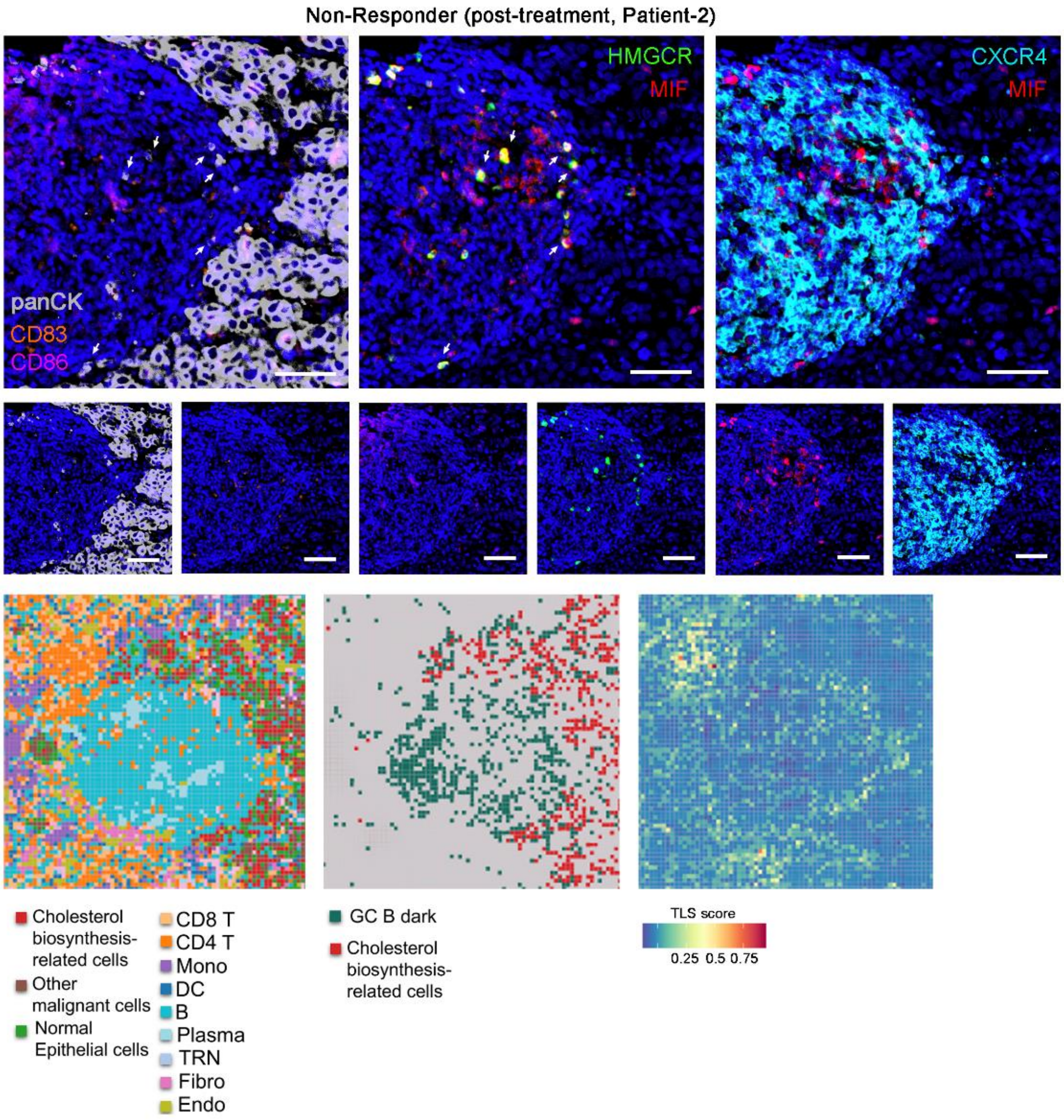

Figure S10. **Representative images of multiplex immunohistochemistry (mIHC) in the non-responder post-treatment sample**. It shows the expression and colocalization of ligand MIF from cholesterol biosynthesis-related tumor cells (high HMGCR and MIF, and positive Pan-CK) and receptor CXCR4 of GC B cells (high CXCR4, low CD83 and CD86). Scale bar: 100 μm. The bottom three images show spatial transcriptome data (8 × 8 μm) for the same region with above mIHC assay. The corresponding H&E staining of the same region was shown in Figure 3d ROI 1. Left: cell type annotations; Middle: Spatial distribution of cholesterol biosynthesis-related tumor cells and GC B dark zone cells; Right: TLS scores. Arrows indicate panCK^{+}HMGCR^{+}MIF^{+} cells.
